# Randomized double-blind clinical study in patients with COVID-19 to evaluate the safety and efficacy of a phytomedicine (P2Et)

**DOI:** 10.3389/fmed.2022.991873

**Published:** 2022-09-08

**Authors:** Claudia Urueña, Ricardo Ballesteros-Ramírez, Alejandra Gomez-Cadena, Alfonso Barreto, Karol Prieto, Sandra Quijano, Pablo Aschner, Carlos Martínez, Maria I. Zapata-Cardona, Hajar El-Ahanidi, Camilla Jandus, Lizdany Florez-Alvarez, Maria Teresa Rugeles, Wildeman Zapata-Builes, Angel Alberto Garcia, Susana Fiorentino

**Affiliations:** ^1^Grupo de Inmunobiologiay Biología Celular, Facultad de Ciencias, Unidad de Investigación en Ciencias Biomédicas, Pontificia Universidad Javeriana, Bogotá, Colombia; ^2^Department of Pathology and Immunology, University of Geneva, Geneva, Switzerland; ^3^Ludwig Institute for Cancer Research, Lausanne Branch, Lausanne, Switzerland; ^4^Oficina de Investigaciones, Hospital Universitario San Ignacio, Bogotá, Colombia; ^5^Departamento de Cardiología, Clínica CardioVID, Medellín, Colombia; ^6^Grupo Inmunovirología, Facultad de Medicina, Universidad de Antioquia UdeA, Medellín, Colombia; ^7^Department of Parasitology, Institute of Biomedical Sciences at the University of São Paulo, São Paulo, Brazil; ^8^Grupo Infettare, Facultad de Medicina, Universidad Cooperativa de Colombia, Medellín, Colombia; ^9^Departamento de Cardiología, Hospital Universitario San Ignacio – Pontificia Universidad Javeriana, Bogotá, Colombia

**Keywords:** COVID-19, polyphenols, immunomodulation, phytomedicine, complementary and alternative medicine, polymolecular drugs

## Abstract

**Background:**

It has been proposed that polyphenols can be used in the development of new therapies against COVID-19, given their ability to interfere with the adsorption and entrance processes of the virus, thus disrupting viral replication. Seeds from *Caesalpinia spinosa*, have been traditionally used for the treatment of inflammatory pathologies and respiratory diseases. Our team has obtained an extract called P2Et, rich in polyphenols derived from gallic acid with significant antioxidant activity, and the ability to induce complete autophagy in tumor cells and reduce the systemic inflammatory response in animal models.

**Methods:**

In this work, a phase II multicenter randomized double-blind clinical trial on COVID-19 patients was designed to evaluate the impact of the P2Et treatment on the clinical outcome and the immunological parameters related to the evolution of the disease. The Trial was registered with the number No. NCT04410510^*^. A complementary study in an animal model of lung fibrosis was carried out to evaluate *in situ* lung changes after P2Et *in vivo* administration. The ability of P2Et to inhibit the viral load of murine and human coronaviruses in cellular models was also evaluated.

**Results:**

Patients treated with P2Et were discharged on average after 7.4 days of admission vs. 9.6 days in the placebo group. Although a decrease in proinflammatory cytokines such as G-CSF, IL-15, IL-12, IL-6, IP10, MCP-1, MCP-2 and IL-18 was observed in both groups, P2Et decreased to a greater extent G-CSF, IL-6 and IL-18 among others, which are related to lower recovery of patients in the long term. The frequency of T lymphocytes (LT) CD3+, LT double negative (CD3+CD4-CD8-), NK cells increased in the P2Et group where the population of eosinophils was also significantly reduced. In the murine bleomycin model, P2Et also reduced lung inflammation and fibrosis. P2Et was able to reduce the viral replication of murine and human coronaviruses *in vitro*, showing its dual antiviral and anti-inflammatory role, key in disease control.

**Conclusions:**

Taken together these results suggest that P2Et could be consider as a good co-adjuvant in the treatment of COVID-19.

**Clinical trail registration:**

https://clinicaltrials.gov/ct2/show/NCT04410510, identifier: NCT04410510.

## Introduction

The COVID pandemic has thrown the world into chaos, destabilizing health systems and leading the world into an unprecedented economic recession. Although the development of vaccines has been rapid and there is evidence of cross-immunity against the new variants, respiratory infections continue to require significant health care.

Phytomedicines have been used in multiple pathologies in which inflammation is principal clinical manifestation such as cancer, diabetes and respiratory diseases (1). The extracts of *Echinacea spp, Sambucus nigra, Pelargoniun sidoides, Curcuma longa* and others decrease the expression of transcription factors related to pro-inflammatory cytokines, increase the activity of CD4 and CD8 TL cells, exhibits immunostimulatory activity, and some of them decrease the risk of developing co-infections during a respiratory disease (2), and the severity of the clinical picture (3). These phytomedicines have a high antioxidant capacity due in part to the polyphenols content that have been linked to their anti-inflammatory action (4, 5). Further, many naturally-occurring metabolites exhibit anti-viral activity against COVID19 by directly binding to viral proteins or by inhibiting the angiotensin-converting enzyme type 2 ACE2 receptor (6).

In respiratory pathologies such as SARS-CoV, MERS-CoV, and COVID-19, an important inflammatory component is observed. Patients infected with COVID-19 have high amounts of IL1-β, IFN-γ, IP-10, IL-18, and MCP-1. In addition, patients requiring intensive care unit (ICU) admission have higher concentrations of G-CSF, IP-10, MCP-1, MIP1, and TNF-α, suggesting that the cytokine storm is associated with the severity of the disease (7, 8). The inhibition of NF-kB by polyphenols might influence the suppression of the inflammation through the inhibition of more than 150 stimuli including cytokines (IL-1β, IL-6, TNF-α, GM-CSF, MCP-1) and TLRs, among others (9).

The standardized extract called P2Et, rich in polyphenols, is highly antioxidant, decreases lipid peroxidation and tissue damage and induces complete autophagy in tumor or stressed cells (10, 11). The induction of a complete autophagic flux could inhibit the replication of beta coronaviruses such as SARS-CoV-2 and increase viral clearance reducing secondary effects of the infection as recently proposed (12). Moreover, P2Et decreases NFκB-dependent pro-inflammatory cytokine production, such as IL-6 (13). This background suggests that the treatment of COVID-19 patients with the P2Et extract could improve their general condition and decrease inflammatory mediators and viral load. To test this hypothesis, a multicenter double-blind randomized controlled clinical trial was designed in COVID19 patients who were admitted in two Colombian hospitals. Clinical and immunological parameters were analyzed after P2Et treatment, and a complementary study in an animal model of lung fibrosis was carried out to evaluate *in situ* lung changes after P2Et treatment.

## Materials and methods

### Clinical study

#### Study patients and inclusion/exclusion criteria

The clinical study was conducted aligned with the Helsinki Declaration, Good Clinical Practice (GCP) and local regulations (14, 15). The study was reviewed and approved by the independent ethics committees (IECs) of the Hospital Universitario San Ignacio (Approval: 2020/050) and Centro Cardiovascular Colombiano Clínica Santa María (Approval: 2021/177). Trial was registered with the number No. NCT04410510 (https://clinicaltrials.gov/ct2/show/NCT04410510). The study is a multi-centered randomized double blind clinical trial conducted at the Hospital Universitario San Ignacio in Bogotá, Colombia and Centro Cardiovascular Colombiano Clínica Santa María (CardioVID) in Medellín, Colombia. The patients were recruited from different departments of the hospitals.

Patients who had a positive PCR COVID19 test and met one of the following criteria were evaluated for eligibility: (1) Hypoxemia with additional oxygen requirements (16), (2) Severe pneumonia - suspected respiratory infection or/and organ failure, (3) SaO_2_ <90% or respiratory rate > 30 breaths/min (17), (4) acute respiratory distress syndrome (ARDS) (16), (5) sepsis, septic shock or mechanically ventilated at the ICU (18). Additionally, eligible female subjects should not be pregnant or lactating and if they were of childbearing age, they would have been using at least one effective contraceptive method.

The exclusion criteria were: (a) Negative laboratory diagnostic test for COVID-19 (PCR COVID-19), (b) Pregnancy or lactation (c) History of allergic reactions attributed to polyphenol-type compounds similar to those found in green tea (d) Psychiatric disorders or mental conditions that are expected to interfere with the purposes of the study (e) Patients undergoing renal replacement therapy in any modality (f) Patients with a prolonged QTc interval, defined as greater than 500 msec (g) Patients with Child C liver cirrhosis or liver failure (h) Patients with elevated Glutamic oxaloacetic transaminase (TGO), TGO 3 times above the 99th percentile of the normal range of the reference laboratory (i) Recipients of solid organ or hematopoietic component transplants (j) Patients undergoing chemotherapy treatment during hospitalization (k) The use of vitamins or herbal supplements must have been suspended at least two weeks prior to study entry, these products refer to herbal supplements like iscador, veregen or mytesi and mixture of vitamins. Therefore, the consumption of vitamin D and vitamin C would not affect study enrollment.

### Study design and sample size

The CS002-COVID19 protocol was a randomized, double-blind, placebo-controlled study that sought to demonstrate the efficacy and safety of treatment with P2Et in patients with a clinical diagnosis of COVID19 infection in addition to standard care. These standards consisted of respiratory support according to severity, use of corticosteroids and supportive therapy according to each clinical case (19). The sample size was calculated in 100 subjects (50 in each group), considering that a difference in the main objective of 2 days of hospital stay between the groups would be optimal to define if there was differential activity. The median hospital stay of patients with COVID19 infection was 7 days with a variability of 3 days (SD 3). Therefore, the sample was calculated to obtain a significant superiority with an alpha level = 0.05 and with a power of 90%.

### Randomization and blinding strategy

Simple non-stratified randomization was implemented using a random number generator that was loaded into the Electronic Case Report Form (eCRF). After the enrollment of the patient, the pharmacist who was the only non-blinded study staff, performed the randomization and according to the allocation of the patient, the treatment or placebo was dispensed.

The pills, bottles, and labels for the placebo and the P2Et were identical, and the only difference was the number of the bottle. The pharmacist was the only study staff personal who knew if the subject was receiving placebo or P2Et.

### P2Et herbal extract

Pods of *Caesalpinia spinosa* (Molina) Kuntze (Divi-divi or tara) were collected in Villa de Leyva, Boyacá, Colombia and identified by Carlos Alberto Parra of the National Herbarium of Colombia (copy number of voucher COL 588448). The P2Et is produced as described from the pods of *C. spinosa* (20, 21). This herbal drug is manufactured in a validated production process under GMP conditions in LABFARVE laboratories. The standardization and manufacturing of the P2Et was carried out according to the FDA Guidance for Herbal Drugs (22) and WHO (world Health Organization) regulations for pharmaceutical and herbal medicines. The batch-to-batch consistency for P2Et is guaranteed (23).

In preclinical studies, P2Et did not show genotoxic or mutagenic effects (20), likewise in acute oral toxicity and sub-chronic toxicity studies (28 days), no clinical alterations were observed (24). The P2Et standardized extract has a low inhibitory and inducing potential for cytochrome P450 (CYP450) enzymes. Therefore, pharmacokinetic interactions with drugs or phytotherapeutics are not expected (data not published). The pharmacokinetic single dose study in rats showed plasma concentration of the chemical markers of P2Et [at 400 mg/Kg gallic acid (468.57 ng/mL), methyl gallate (19.63 ng/mL) and ethyl gallate (337.47 ng/mL)] with increasing dose levels in *Sprague Dawley* rats. The 3 marker components for the P2Et are eliminated at 24 h and no clinical symptoms of toxicity were observed in any of the study groups at the doses used (data not published). In the phase I clinical trial, P2Et was safe for use in healthy humans with a maximum tolerated dose of 600 mg/d. There was no severe toxicity, only mild adverse events for the most part, with no significant out-of-range changes in the safety parameters (24).

In this trial, the P2Et was used in a dosage capsule of 250 mg of active ingredient with starch as excipient to be taken twice a day for 14 days; the placebo was manufactured under the same conditions without extract and storage in the same conditions as P2Et pills. The presentation of extract was a bottle with 28 capsules, with a label according to requirements of the local regulation for clinical trials. The investigational product was stored in the research center from Hospital Universitario San Ignacio at a temperature below 30°C and humidity below 70.0 guarantying the stability of the product.

### Safety and efficacy assessment

Adverse events, tolerability and safety were evaluated based on the National Institute of Health Common Terminology Criteria for Adverse Events (CTCAE) version 4.0 (25) once a day for 14 days, which included clinical follow-up, measure vital signs and classification in the six modified category ordinal scale recommended by the WHO R&D Blueprint expert group. The six modified category ordinal scale consisted of the following categories: 1 - not hospitalized; 2 - Hospitalized, but not requiring supplemental oxygen; 3 - Hospitalized and requiring supplemental oxygen; 4 - Hospitalized and requiring high-flow nasal oxygen or non-invasive mechanical ventilation; 5 - Hospitalized requiring extracorporeal membrane oxygenation, invasive mechanical ventilation, or both; and 6 – Death. This scale allows the classification of patients according to infection severity. Clinical improvement was considered when the patient drops at least one point on the scale and clinical worsening was considered when the patient raised at least one point on the scale. Grading was done daily by the medical staff.

The clinical laboratory tests were performed at baseline and in the day 14 after taken the last pill. Safety assessments included kidney (creatinine, blood ureic nitrogen), hepatic (alkaline phosphatase, aspartate aminotransferase, alanine aminotransferase, total bilirubin, direct bilirubin, indirect bilirubin, lactate dehydrogenase, total proteins) electrolyte (sodium, potassium, calcium, magnesium), metabolic (glycemia, cholesterol, high-density lipoprotein, low-density lipoprotein, triglycerides, uric acid, albumin) and hematological (hemoglobin, hematocrit, platelets, erythrocytes, leukocytes) parameters. All adverse events that occurred during the trial were recorded along with the duration, severity, and relationship to the P2Et. The tolerability and safety profile were defined by the principal investigators after analyzing the clinical results of the exams.

The effectiveness was evaluated as the length of hospital stay, counted from the day of admission until the day of discharge (inclusive). For the assessment of the main objective, the length of hospital stay was quantified. The initial day was defined as the first day of the first dose and ended at the time of hospital discharge. For the assessment of aims associated with clinical improvement (Secondary Objectives) the modified ordinal scale of six categories was used.

### Antiviral activity assay

*In vitro* P2Et antiviral activity experiments used the *Cercopithecus aethiops* kidney cell line Vero E6 (provided by Instituto Nacional de Salud, Colombia). Infections were carried out with a Colombian SARS-CoV-2 isolate of the D614G ancestral strain (EPI_ISL_536399) (22). The cytotoxicity of P2Et (6.25 - 200 μg/mL) was assessed using the MTT [(3-(4,5-dimethylthiazol-2-yl)-2,5-diphenyltetrazolium bromide] assay and the antiviral activity was evaluated by a pre-post-infection treatment strategy, as was previously described (26, 27). The infectious viral particles of treated and untreated cultures (infection control) were quantified by plaque assay and compared to calculate the infection percentages (*n* = 2). Chloroquine (100 μM) was used as a positive control of viral inhibition. The supernatant of infected cells without treatment was used as infection control. Two independent experiments with two replicate each were performed (*n* = 4).

The antiviral activity of P2Et was determined by quantifying the infectious viral particles of SARS-CoV-2 in supernatants of treated cells using the plaque assay. Briefly, 1.2 x 10^5^ Vero E6 cells/well were seeded in 24-well plates for 24 h, at 37°C, with 5% CO_2_. Subsequently, 10–fold serial dilutions of the supernatants obtained from the antiviral assay (200μL/well) were added to cell monolayers and incubated for 1 h at 37°C with 5% CO_2_. Then, the viral inoculum was removed and replaced by 1 mL of semi-solid medium (1.5% Carboxymethyl-cellulose in DMEM 1X with 2% FBS and 1% Penicillin-Streptomycin). Cells were incubated for 3–4 days at 37°C with 5% CO_2_. Finally, monolayers were washed twice with PBS (phosphate buffered-saline), fixed/stained with 4 % Formaldehyde/ 1% Crystal violet solution, and viral plaques were counted. The difference between the viral titer after treatment and the infection control was expressed as inhibition percentage (*n* = 2).

### Patient's sample collection

**Figure 1:**
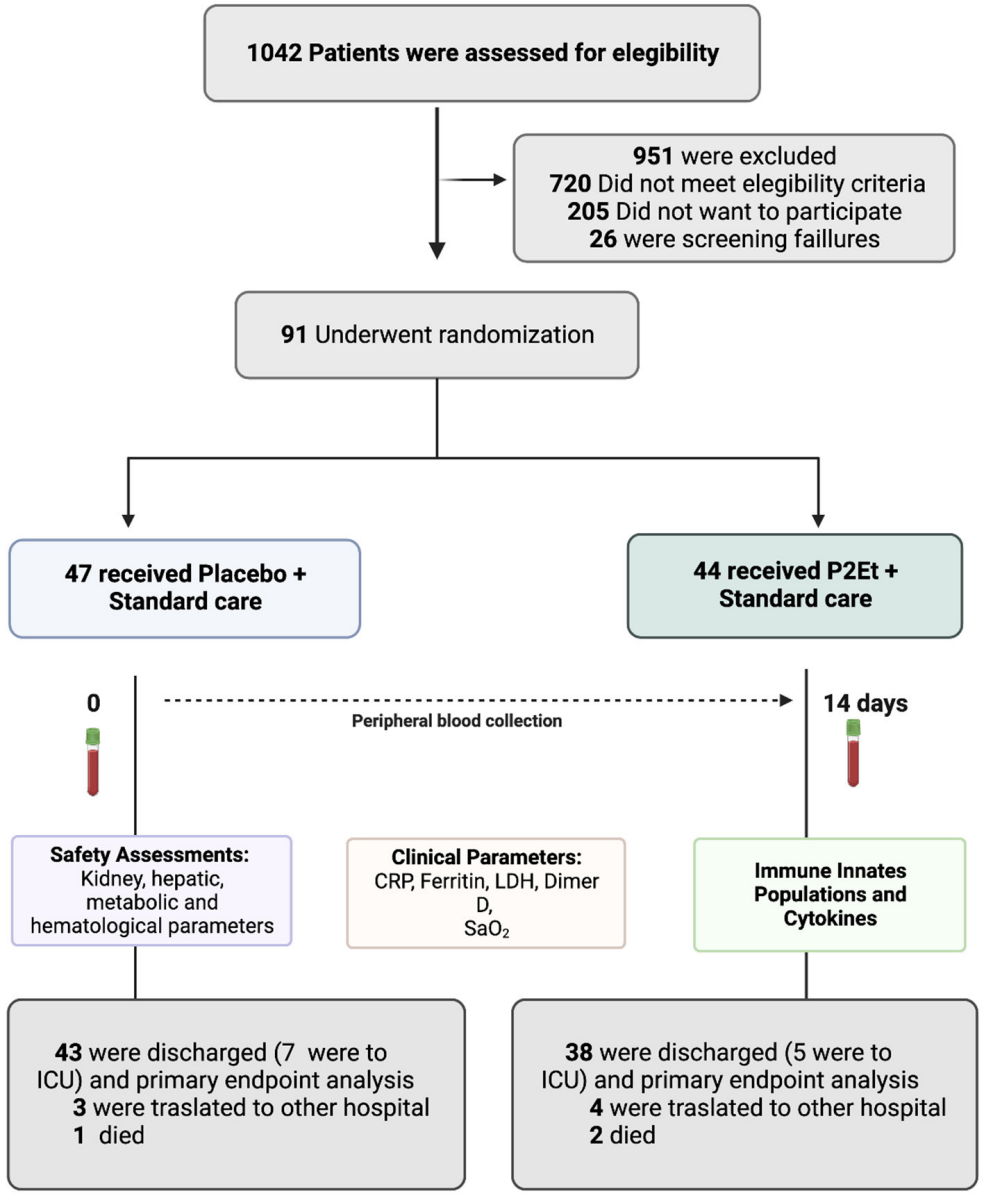
Screening, Randomization, and Outcomes. COVID-19 patients were included in the study. 1,042 patients were screened and 91 patients underwent randomization. 47 patients received placebo and 44 patients received P2Et extract. A blood sample was taken from each patient before and after treatment to assess safety parameters, clinical parameters, immune innate cell populations by flow cytometry and cytokines for a MAGPIX^®^ System. This figure was created using BioRender (https://Biorender.com/).

Peripheral blood (PB) samples from patients were collected on day 1 and day 14 for the identification and quantification of leukocyte subpopulations (Figure 1). All patients provided written informed consent to participate in the study. For Innate Lymphoid cells (ILCs) analysis, peripheral blood mononuclear cells (PBMCs) were isolated by density-gradient centrifugation using Ficoll-PaqueTM PREMIUM (GE Healthcare, Chicago, Illinois, United States). A total of 1 x 10^7^ cells were cryopreserved in liquid nitrogen in freezing media (RPMI-1640 50%, FBS 40%, and 10% DMSO) until use for flow cytometry characterization. Serum from patients was collected and conserved at−80°C for cytokine analyses (Figure 1).

### Measurement of serum cytokine levels using multiplex immunoassays

To measure cytokines in human serum (G-CSF, IFN-γ, IL-10, IL-13, IL-15, IL-2, IL1-RA, IL-4, IL-5, IL-6, IL-7, IL-8, IP-10, MCP-1, MIP1-α, TNF-α, MCP-2, TSLP, IL-33, NTproBNP, IL-14, IL-18, IL-22, and IL-17E) of COVID-19 patients (day 1 and day 14) different commercially available multiplex immunoassay were used: (MILLIPLEX MAP Human Cytokine/Chemokine Magnetic Bead Panel Immunology and Panel II Immunology Multiplex Assay; MILLIPLEX MAP Human Cardiovascular Disease (CVD) Magnetic Bead Panel 1, Cardiovascular Multiplex Assay; MILLIPLEX MAP Human TH17 Magnetic Bead Panel-Immunology Multiplex Assay; MILLIPLEX MAP Human Cytokine/Chemokine Magnetic Bead Panel IV; MILLIPLEX Human Cytokine/Chemokine/Growth Factor Panel A-Immunology Multiplex Assay, Millipore, Burlington, MA, United States). The assays were performed in 96-well plates according to the manufacturer's instructions and the results were expressed in pg/mL. Serum samples (25 μL) were added into the wells with 25 μL of assay buffer, followed by the addition of matrix solution. After mixing, 25 μL of beads were added to the wells, and plates were incubated overnight at 4°C with shaking. After incubation, fluid was removed, and plates were washed. After addition of 25-μL detection antibodies, plates were incubated for 1 h at room temperature with shaking. Then, 25 μL of streptavidin-phycoerythrin was added to each well, and plates were further incubated for 30 min at room temperature with shaking. Fluid was removed, and plates were washed. Then, 150 μL of sheath fluid was added. After resuspension for 5 min, the median fluorescent intensities were determined on a MAGPIX^®^ System. Cytokine concentrations were calculated through the five-parameter logistic curve-fitting method using the median fluorescence intensity.

To measure cytokines in the animal model 2 different commercially available bead-based multiplex immunoassays were used according to manufacturer's recommendations: LEGENDplex^TM^ mouse Th cytokine panel (IFN-γ, IL-5, TNF-α, IL-2, IL-6, IL-4, IL-10, IL-9, IL-17A, IL17-F, IL-22, IL-13) and mouse cytokine panel 2 (IL-1a, IL-1b, IL-3, IL-12p40, IL12-p70, IL-23, IL-7, IL-11, IL-27, IL-33, IFN-β, GM-CSF, TSLP). Cytokine concentrations were calculated using the QOGNIT LEGENDplex online software (https://legendplex.qognit.com/user/login?next=workflow.workflow) and are reported in pg/ml.

### Flow cytometry

The PBMCs were thawed at 37°C and washed with PBS 1X. Cells were stained with the green viability dye for 30 min at 4°C in the dark, then washed with FACS buffer and stained with the antibody mix for 30 min at 4°C in the dark. The antibody mix was composed as follow: FITC Lineage [CD3 (REA613, Miltenyi Biotech, 1/800), CD4 (REA623, Miltenyi Biotech, 1/100), CD14 (REA599, Miltenyi Biotech, 1/400), CD15 (VIMC6, Miltenyi Biotech, 1/800), CD19 (REA675, Miltenyi Biotech, 1/400), CD20 (REA780, Miltenyi Biotech, 1/800), CD33 (REA775, Miltenyi Biotech, 1/200), CD34 (AC136, Miltenyi Biotech, 1/200), CD203c (Invitrogen, 1/100), FceRIa (REA758, Miltenyi Biotech, 1/400)], PE-CD8 RPA-T8, Biolegend, 1/200, PE-Dazzle-CD127 (A019D5, Biolegend, 1/400), PercP-Cy5.5-NKp46 (9E2, Biolegend, 1/200), PE-Cy7-KLRG1(13F12F2, e-Biosciences, 1/50), BV421-CD294 (BM16, Becton Dickinson, 1/50), BV786-CD94 (HP-3D9, Becton Dickinson, 1/200),BV510-CD25 (BC96, Biolegend, 1/100), BV570-CD56 (HCD56, Biolegend, 1/50),BV605-CD117 (104D2, Biolegend, 1/200), BV650-CD69 (FN50, Biolegend, 1/200), BV711 anti-human PD1(EH12.2H7, Biolegend, 1/400), APC -CD278 (C398.4A, Biolegend, 1/50), Alexa Fluor 700-CD16 (3G8, Biolegend, 1/100) and APCeF780-CD27(O323, e-Biosciencies, 1/100). Then, cells were acquired using Cytek Aurora flow cytometer (Cytek Biosciences, Fremont, CA), and the results were subsequently analyzed using SpectroFlo^®^ software V 3.0 (Cytek Biosciences, Fremont, CA), and FlowJo 10.8.1 Software (Tree star, Ashland, OR). Representative gating strategy is included in Supplementary Figure 2.

For the identification and quantification of leukocyte subpopulations in patients' PB, whole blood was labeled using the commercial LST (Lymphoid Screening Tube) insert (Cytognos-SL) standardized by the European EuroFlow Consortium which includes a mixture of 12 antibodies combined in 8 different fluorescences (28) for the following markers: Pacific Blue-CD20 (2H7), Pacific Blue-CD4 (RPA-T4), OC515-CD45 (GA90), FITC-CD8 (UCHT-4), SIgKappa (polyclonal), PE-CD56 (C5.9), SIgLambda (polyclonal), PE-Cy7-CD19 (SA287), TCR**γδ** (11F2), PercpCy5.5-CD5 (L17F12), APC-CD3 (UCHT-1), and APC-750-CD38 (LD38). For membrane antigen labeling, 300 μl of PB sample was incubated with the antibodies for 30 min at room temperature in the dark. After this time, 2 mL of lysis solution (FACS lysing solution-BDB) was added for 15 min and two washes were performed with 2 mL of PBS, followed by centrifugation for 5 min at 2,000 rpm. Then, the cells were acquired by flow cytometry using a FACSAria IIU flow cytometer (BD Immunocytometry Systems, San José, CA, United States), and the results were subsequently analyzed using Infinicyt software (Cytognos SL).

Murine ILCs were identified as CD45^+^Lin^−^CD90.2^+^ among alive cells. Lineage markers, all FITC conjugated, include: CD3e (17A2, in house, 1:200), DX5 (DX5, Miltenyi Biotec, 1:200), CD5 (53.7, in house, 1:200), CD19 (ID3, in house, 1:200), CD11b (M1/70, in house, 1:200), CD11c (N418, in house, 1:200), B220 (RA3-6B2, in house, 1:200), FCεRIα (MAP-1, Miltenyi Biotec, 2:50), Ter119 (Ter119, in house, 1:200), TCRγδ (2M31/11, in house, 1:200), and TCRαβ (H57, in house, 1:200). Additional markers used to identify the ILC subpopulations include: PE-ST2 (RMST2-2, Invitrogen, 1:200), PE-Cy7-KLRG1 (2F1/KLRG1, Biolegend, 1:200), BV711-NK1.1 (PK136, Biolegend, 1:100), Brilliant Violet 605-CD90.2 (53-2.1, Biolegend, 1:1,000), Alexa-Fluor 700-CD45.2 (AL1-4A2, in house, 1:400), BV650-NKp46 (29A1.4 Biolegend, 1:200), BUV395-CD4 (GK1.5, BD, 1:200), BV421-DX5 (DX5, Invitrogen, 1:200), BV510-CD69 (H1.2F3, Biolegend, 1:200), APC-Cy7-PD1 (29F1.A12, Biolegend, 1:200), BV785-CCR6 (29-2L17, Biolegend, 1:200), PECy5-CD3 (145-2C11, Biolegend, 1:300). Representative gating strategy is included in Supplementary Figure 4. Intracellular staining was performed after fixation and permeabilization using the Foxp3 fix/perm buffer set Biolegend according to the manufacturer recommendations (Biolegend, 421403). For the intracellular staining the following antibodies were used: PerCPCy5.5 anti-mouse GATA3 (TWAJ, Invitrogen, 1:100), APC anti-mouse T-bet (4B10, Biolegend, 1:100), PE-efluor610 anti-mouse RORgt (B2D, Invitrogen, 1:100), Brilliant Violet 421 anti-mouse IL-5 (TRFK5, Biolegend, 1:50), and PEefluor610 anti-mouse IL-13 (4311635, eBioscience, 1:100).

### Bleomycin animal model

Female WT C57BL/6J 6 weeks old mice were purchase by Envigo. Animals were challenged with 2U/kg of bleomycin (Bleomycin Sulfate, Streptomyces verticillus, Merck Ref 203401) intranasally in a total volume of 20μL. 1 day after challenge, animals were treated either with PBS as a negative control or with the P2Et solution. Each animal received 37,5 mg/kg per mouse per dose in a total volume of 100μL by i.p injection every 2 days. The weight of the animals was monitored also every 2 days. All the groups were euthanized by i.p injection of pentobarbital at day 8 post-challenge. Lungs and blood were collected for cellular and soluble factors analysis. This study was approved by the Veterinary Authority of the Swiss Canton Genève (authorization no. GE119/20) and performed in accordance with Swiss ethical guidelines. All animals were maintained at the University of Geneva's animal facility under a 12 h dark/light cycle, at 21°C ± 1°C and 55% ± 10% HR.

### QPCR for fibrosis quantification in animal model

Total RNA was isolated from lung tissue snap-frozen and stored at−80°C using the TRIZOL reagent according to the manufacturer's instructions (Invitrogen cat 15596026). Final preparation of RNA was considered DNA- and protein-free if the ratio of readings at 260/280 nm was ≥1.7. Isolated mRNA was reverse-transcribed by the “High-Capacity cDNA Reverse Transcription Kit” (Thermofisher cat 4368814) according to manufacturer's recommendations. The quantitative real-time PCR was carried out in the Applied Biosystems 7900HT Fast Real-Time PCR Sequence Detection System (Applied Biosystems) with specific primers: TNFα forward 5'-GCACCACCATCAAGGACTCA-3' reverse 5'-GAGACAGAGGCAACCTGACC3-'; TGFB1 forward 5'-CTT CAA TAC GTC AGA CAT TCG GG-3' reverse 5'-GTA ACG CCA GGA ATT GTT GCT A-3'; MMP19 forward 5'-GCT GAC ATT CGC CTC TCT TTC-3' reverse 5'-CAC TCC TTG ATA GGT CCC CTC-3'; LOXL1 forward 5'-TGC CCG ACA ACT GGA GAG A-3' reverse 5'-TGC GGA TAG GGG AAC TGC T-3'; HPRT1 forward 5'-CCCAGCGTCGTGATTAGTGATG-3' reverse 5'- TTCAGTCCTGTCCATAATCAGTC-3' using KAPA SYBR^®^ FAST qPCR Kits (cat KK4602 Roche). Samples were amplified simultaneously in triplicate in one-assay run with a non-template control blank for each primer pair to control for contamination or for primer dimerization, and the Ct value for each experimental group was determined. The housekeeping gene HPRT1 Hypoxanthine phosphoribosyltransferase (1) was used as an internal control to normalize the Ct values, using the 2–ΔCt formula.

### Statistical analysis

For human samples, the software SPSS version 26 (IBM Corp. Released 2019. IBM SPSS Statistics for Macintosh, Version 26.0. Armonk, NY: IBM Corp) was used for data analysis. A univariate analysis was carried out, reporting qualitative variables through absolute and relative frequencies; in quantitative variables, measures of central tendency and dispersion were done; coefficient of variation in variables with normal distribution according to the Kolmogorov-Smirnov test were used. Those variables that did not have a normal distribution were expressed as the median and the interquartile range with a 95.0% confidence interval.

Log-rank test was used for the primary endpoint and the Chi-Square test for the secondary endpoints. For normal quantitative variables like the leukocyte subpopulations in PB (absolute, percentage and delta), the *t*-test was used for the comparison of averages; for those without normal distribution, the Mann Whitney *U*-test was used. A *p* value of <0.05^*^ was considered statistically significant.

Data from ILCs analysis was analyzed using GraphPad Prism v9.3.1 for Mac OS X (GraphPad Software, La Jolla California United States, www.graphpad.com). Statistical analysis of the significance between two groups was calculated using the Mann–Whitney *U* test. Differences among subject groups were evaluated using Kruskal-Wallis and Dunn's posttest for multiple comparisons. For all cases, the differences were considered statistically significant when p < 0.05.

Data from antiviral activities were analyzed with GraphPad Prism (La Jolla, CA, United States). Data were presented as mean ± SEM. Statistical differences were evaluated by Student's *t*-test or Mann–Whitney *U* test based on the Shapiro Wilk normality test. A *p*-value ≤ 0.05 was considered significant, with ^**^ *p* ≤ 0.01 and ^****^ *p* < 0.0001. The EC50 (median effective concentration) values represent the concentration that reduces viral titer by 50%. The CC50 (half-maximal cytotoxic concentration) values represent the concentration that causes 50% toxicity on Vero E6. The corresponding dose-response curves were fitted by non-linear regression analysis using a sigmoidal model. The calculated selectivity index (SI) represents the ratio of CC50 to EC50.

Data from the bleomycin animal model was analyzed using GraphPad Prism. Data presented shows box and whiskers graphs with min to max representation. Each dot represents one mouse. Statistical differences were evaluated by Kruskal-Wallis test and Dunn's multiple comparisons test or Two-way ANOVA with a Sídák correction for multiple comparisons. In both cases with a familly-wise alpha threshold and confidence level of 0.05 (95% confidence interval). A *p*-value ≤ 0.0332 was consider significant with ^*^, ≤ 0.0021 with ^**^, ≤ 0.0002 with ^***^, ≤ 0.0001 with ^****^.

## Results

### Clinical results

#### Subject characteristics

Between October 1, 2020, and August 27, 2021, a total of 91 patients were enrolled and randomized. 44 out the 91 were assigned to the P2Et arm and 47 to the placebo arm (Figure 1). Demographic and clinical history were recorded for each group, no statistical differences were found between them. Tables 1, 2 summarize the main baseline characteristics of the patients in each group.

**Table 1:** Demographic and clinical history for the patients enrolled in CS002-COVID19.

**Demographic characteristics**	**Placebo** **(*n* = 47)**	**P2Et** **(*n* = 44)**	**Total** **(*n* = 91)**
**Age (years)**			
Mean (SD)	52.9 (14.3)	50.0 (13.4)	51.5 (13.9)
**Sex**			
Women	26 (55.3%)	21 (47.7%)	47 (51.6%)
Men	21 (44.7%)	23 (52.3%)	44 (48.4%)
**Weight (kilograms)**			
Mean (SD)	76.0 (13.4)	79.1 (15.6)	77.5 (14.5)
**Size**			
Mean (SD)	1.63 (0.0849)	1.66 (0.0925)	1.64 (0.0899)
**Systolic pressure (mmHg)**			
Mean (SD)	115 (12.1)	116 (14.8)	116 (13.4)
**Diastolic pressure (mmHg)**			
Mean (SD)	69.7 (7.41)	70.3 (8.14)	70.0 (7.74)
**heart rate (beats per min)**			
Mean (SD)	72.5 (10.1)	73.6 (13.5)	73.0 (11.8)
**Respiratory rate (breaths per minute)**			
Mean (SD)	20.1 (1.56)	20.1 (2.52)	20.1 (2.07)
**SaO2 (%)**			
Mean (SD)	90.7 (2.95)	89.6 (6.93)	90.2 (5.26)
**Head and neck**			
Normal	45 (95.7%)	44 (100%)	89 (97.8%)
Abnormal	2 (4.3%)	0 (0%)	2 (2.2%)
**Cardiovascular**			
Normal	46 (97.9%)	42 (95.5%)	88 (96.7%)
Abnormal	1 (2.1%)	2 (4.5%)	3 (3.3%)
**Pulmonary**			
Normal	41 (87.2%)	42 (95.5%)	83 (91.2%)
Abnormal	6 (12.8%)	2 (4.5%)	8 (8.8%)
**Abdomen**			
Normal	38 (80.9%)	40 (90.9%)	78 (85.7%)
Abnormal	9 (19.1%)	4 (9.1%)	13 (14.3%)
**Extremities**			
Normal	45 (95.7%)	44 (100%)	89 (97.8%)
Abnormal	2 (4.3%)	0 (0%)	2 (2.2%)
**Neurological**			
Normal	46 (97.9%)	44 (100%)	90 (98.9%)
Abnormal	1 (2.1%)	0 (0%)	1 (1.1%)
**Pregnancy test**			
Positive	0 (0%)	0 (0%)	0 (0%)
Negative	18 (38.3%)	12 (27.3%)	30 (33.0%)
**Clinical history**			
**Myocardial infarction**			
Yes	0 (0%)	0 (0%)	0 (0%)
No	47 (100%)	44 (100%)	91 (100%)
**Congestive heart failure**			
Yes	1 (2.1%)	0 (0%)	1 (1.1%)
No	46 (97.9%)	44 (100%)	90 (98.9%)
**Peripheral vascular disease**			
Yes	1 (2.1%)	0 (0%)	1 (1.1%)
No	46 (97.9%)	43 (97.7%)	89 (97.8%)
Missing	0 (0%)	1 (2.3%)	1 (1.1%)
**Cerebrovascular disease**			
Yes	0 (0%)	0 (0%)	0 (0%)
No	47 (100%)	44 (100%)	91 (100%)
**Dementia**			
Yes	0 (0%)	0 (0%)	0 (0%)
No	47 (100%)	44 (100%)	91 (100%)
**Chronic lung disease**			
Yes	1 (2.1%)	0 (0%)	1 (1.1%)
No	46 (97.9%)	44 (100%)	90 (98.9%)
**Connective tissue pathology**			
Yes	0 (0%)	0 (0%)	0 (0%)
No	47 (100%)	44 (100%)	91 (100%)
**Ulcer disease**			
Yes	0 (0%)	0 (0%)	0 (0%)
No	47 (100%)	44 (100%)	91 (100%)
**Mild liver disease**			
Yes	0 (0%)	0 (0%)	0 (0%)
No	47 (100%)	44 (100%)	91 (100%)
**Moderate or severe liver disease**			
Yes	0 (0%)	0 (0%)	0 (0%)
No	47 (100%)	44 (100%)	91 (100%)
**Diabetes**			
Yes	6 (12.8%)	5 (11.4%)	11 (12.1%)
No	41 (87.2%)	39 (88.6%)	80 (87.9%)
**Diabetes with organic damage**			
Yes	0 (0%)	0 (0%)	0 (0%)
No	47 (100%)	44 (100%)	91 (100%)
**hemiplegia**			
Yes	0 (0%)	0 (0%)	0 (0%)
No	47 (100%)	44 (100%)	91 (100%)
**Renal pathology (moderate or severe)**			
Yes	0 (0%)	0 (0%)	0 (0%)
No	47 (100%)	44 (100%)	91 (100%)
**Neoplasms**			
Yes	0 (0%)	0 (0%)	0 (0%)
No	47 (100%)	44 (100%)	91 (100%)
**Leukemias**			
Yes	0 (0%)	0 (0%)	0 (0%)
No	47 (100%)	44 (100%)	91 (100%)
**Malignant lymphomas**			
Yes	0 (0%)	0 (0%)	0 (0%)
No	47 (100%)	44 (100%)	91 (100%)
**Solid metastasis**			
Yes	0 (0%)	0 (0%)	0 (0%)
No	47 (100%)	44 (100%)	91 (100%)
**AIDS**			
Yes	0 (0%)	0 (0%)	0 (0%)
No	47 (100%)	44 (100%)	91 (100%)
**Other**			
Yes	47 (100%)	44 (100%)	91 (100%)
No	0 (0%)	0 (0%)	0 (0%)
**Alcohol**			
Yes	2 (4.3%)	4 (9.1%)	6 (6.6%)
No	45 (95.7%)	40 (90.9%)	85 (93.4%)
**Drugs**			
Yes	0 (0%)	0 (0%)	0 (0%)
No	47 (100%)	44 (100%)	91 (100%)
**Smoke status**			
Nowadays	1 (2.1%)	2 (4.5%)	3 (3.3%)
Previously	7 (14.9%)	8 (18.2%)	15 (16.5%)
Never	39 (83.0%)	34 (77.3%)	73 (80.2%)

**Table 2:** Clinical characteristics of the patients enrolled in CS002-COVID19.

**Parameters**	**Placebo (*n* = 47)**	**P2Et (*n* = 44)**	**Total (*n* = 91)**
**Glucose (mg/dl)**			
Mean (SD)	125 (43.3)	127 (53.5)	126 (48.2)
**Total colesterol (mg/dl)**			
Mean (SD)	159 (39.6)	161 (41.1)	160 (40.1)
**HDL (mg/dl)**			
Mean (SD)	30.2 (7.97)	29.2 (7.15)	29.7 (7.56)
**LDL (mg/dl)**			
Mean (SD)	88.3 (30.3)	95.1 (33.3)	91.7 (31.8)
Missing	1 (2.1%)	0 (0%)	1 (1.1%)
**Triglycerides (mg/dl)**			
Mean (SD)	205 (98.1)	185 (75.8)	195 (88.1)
**Urine analysis**			
Normal	37 (78.7%)	28 (63.6%)	65 (71.4%)
Abnormal	10 (21.3%)	16 (36.4%)	26 (28.6%)
**Blood urea nitrogen (BUN) (mg/dl)**			
Mean (SD)	15.9 (7.42)	16.9 (5.88)	16.4 (6.70)
**Serum creatinine (mg/dl)**			
Mean (SD)	0.748 (0.197)	0.807 (0.206)	0.777 (0.203)
**Uric acid (mg/dl)**			
Mean (SD)	3.66 (1.05)	3.73 (1.23)	3.69 (1.14)
**Alkaline phosphatase (U/L)**			
Mean (SD)	80.0 (33.7)	79.5 (24.5)	79.8 (29.4)
Median [Min, Max]	70.0 [37.0, 199]	78.0 [42.0, 157]	73.0 [37.0, 199]
Missing	1 (2.1%)	0 (0%)	1 (1.1%)
**AST (U/L)**			
Mean (SD)	40.6 (20.5)	39.4 (22.5)	40.0 (21.4)
Missing	1 (2.1%)	0 (0%)	1 (1.1%)
**ALT (U/L)**			
Mean (SD)	49.4 (30.9)	48.2 (30.8)	48.8 (30.7)
Missing	1 (2.1%)	0 (0%)	1 (1.1%)
**Lactate dehydrogenase (LDH) (U/L)**			
Mean (SD)	343 (143)	337 (102)	340 (124)
**Total bilirubin (mg/dl)**			
Mean (SD)	0.543 (0.260)	0.659 (0.344)	0.599 (0.308)
**Direct bilirubin (mg/dl)**			
Mean (SD)	0.141 (0.147)	0.171 (0.207)	0.155 (0.178)
**indirect bilirubin (mg/dl)**			
Mean (SD)	0.403 (0.156)	0.487 (0.190)	0.444 (0.177)
**Sodium (mmol/L)**			
Mean (SD)	139 (2.90)	138 (3.23)	138 (3.08)
**Potassium (mmol/L)**			
Mean (SD)	4.14 (0.400)	4.10 (0.373)	4.12 (0.386)
**Calcium (mg/dl)**			
Mean (SD)	8.33 (0.421)	8.41 (0.501)	8.37 (0.460)
**Magnesium (mg/dl)**			
Mean (SD)	2.10 (0.231)	2.02 (0.191)	2.06 (0.215)
**Total proteins (g/dl)**			
Mean (SD)	6.05 (0.632)	6.23 (0.760)	6.13 (0.699)
**Albumin (g/dl)**			
Mean (SD)	3.27 (0.351)	3.38 (0.393)	3.32 (0.373)
**Ferritin**			
Mean (SD)	783 (761)	1,130 (907)	952 (848)
**Total erythrocyte count x 10** ^**3**^			
Mean (SD)	4,870 (543)	4,930 (613)	4,900 (575)
**Platelet count x 10** ^**3**^			
Mean (SD)	278 (124)	260 (93.9)	269 (110)
**Hemoglobin (gr/dl)**			
Mean (SD)	13.8 (2.19)	14.5 (1.81)	14.2 (2.03)
**Hematocrit (%)**			
Mean (SD)	41.3 (5.83)	42.7 (5.11)	42.0 (5.51)
**Total white blood cell count x 10** ^**3**^			
Mean (SD)	7,890 (3,810)	8,360 (3,070)	8,120 (3,460)
**Neutrophils absolute value (x10** ^**3**^ **)**			
Mean (SD)	6,470 (3,710)	6,730 (2,930)	6,590 (3,340)
**Neutrophils (%)**			
Mean (SD)	79.4 (10.5)	78.9 (8.49)	79.1 (9.52)
**Lymphocytes absolute value(x10** ^**3**^ **)**			
Mean (SD)	956 (455)	1,080 (484)	1,010 (470)
**Lymphocytes (%)**			
Mean (SD)	14.0 (8.25)	14.2 (6.92)	14.1 (7.60)
**Eosinophils absolute value (x10** ^**3**^ **)**			
Mean (SD)	6.53 (24.7)	4.00 (18.3)	5.35 (21.9)
Missing	0 (0%)	3 (6.8%)	3 (3.3%)
**Eosinophils (%)**			
Mean (SD)	2.29 (14.6)	0.100 (0.190)	1.23 (10.5)
**Basophils absolute value (x10** ^**3**^ **)**			
Mean (SD)	3.51 (15.0)	10.0 (27.3)	6.65 (22.0)
**Basophils (%)**			
Mean (SD)	0.187 (0.124)	0.270 (0.206)	0.227 (0.173)
**Monocytes absolute value (x10** ^**3**^ **)**			
Mean (SD)	424 (202)	479 (265)	451 (235)
**Monocytes (%)**			
Mean (SD)	6.08 (3.05)	6.32 (2.69)	6.20 (2.87)

### Safety

A total of 75 adverse events were observed in the P2Et group, of which 47 were classified as grade 1; 24 as grade 2; 2 as grade 4, and 2 as grade 5. There were 2 deaths unrelated to the treatment in the P2Et arm and 1 death in placebo arm. The characteristics of the reported adverse events are described in Table 3. The detail of this adverse events is described in Supplementary Table 1.

**Table 3:** Characteristics of Adverse Events presented in the P2Et group and placebo arm.

	**Placebo**	**P2Et**	**Overall**
	**(*N* = 84)**	**(*N* = 75)**	**(*N* = 159)**
**Serious adverse event**			
Produced or prolonged hospitalization	0 (0%)	0 (0%)	0 (0%)
Congenital anomaly	0 (0%)	0 (0%)	0 (0%)
Threat to life	6 (7.1%)	2 (2.6%)	8 (5.1%)
Cause death	1 (1.2%)	2 (5.2%)	3 (3.2%)
Produced disability or incapacity/increased/worsened	0 (0%)	0 (0%)	0 (0%)
Not serious	77 (91.7%)	71 (94.8%)	148 (91.7%)
**Severity of the adverse event**			
Grade 1	51 (60.7%)	47 (62.7%)	98 (61.6%)
Grade 2	24 (28.6%)	24 (32.0%)	48 (30.2%)
Grade 3	2 (2.4%)	0 (0%)	2 (1.3%)
Grade 4	6 (7.1%)	2 (2.7%)	8 (5.0%)
Grade 5	1 (1.2%)	2 (2.7%)	2 (1.9%)
**Outcome of the adverse event**			
Recovered without sequel	57 (67.9%)	52 (69.3%)	109 (68.6%)
Recovering with sequel	0 (0%)	1 (1.3%)	1 (0.6%)
Not recovered or not resolved	1 (1.2%)	5 (6.7%)	6 (3.8%)
Death associated with the adverse reaction	0 (0%)	0 (0%)	0 (0%)
Death- drug may have contributed	0 (0%)	0 (0%)	0 (0%)
Death - not drug related	1 (1.2%)	2 (2.7%)	3 (1.9%)
Not known	24 (28.6%)	16 (16.0%)	36 (22.9%)
Missing	1 (1.2%)	3 (4.0%)	4 (2.5%)
**Relationship with drug use**			
Yes	0 (0%)	0 (0%)	0 (0%)
No	62 (73.8%)	60 (80.0%)	122 (76.7%)
Reasonable chance	9 (10.7%)	5 (6.7%)	14 (8.8%)
No reasonable chance	13 (15.5%)	10 (16.0%)	25 (15.7%)

### Primary outcome and secondary outcomes

The median length of hospitalization in the P2Et group was 7.395 days (IC 95% 5.641–9.148) vs. placebo group with a median of 9.581 (IC 95% 7.537–11.626) (*p* value: 0.123) (Supplementary Figure 1). In the secondary outcomes, there were no statistically significant differences between the two groups in patients who achieved clinical improvement according to the modified ordinal scale at day 14 (P2Et: 84.09%—Placebo: 85.10% *p*: 0.561) and day 28 (P2Et: 88.63% —Placebo: 91, 48% *p*: 0.458). Regarding admission to the ICU (P2Et: 11.36%—Placebo: 14.89% *p*: 0.427) and mortality (P2Et: 4.80%—Placebo: 2.22% *p*: 0.435), there were no statistically significant differences between the groups.

### Immune cell modulation

Hemogram findings at 14 days were similar, showing that leukocyte counts normalized in 86.1% of cases treated with P2Et vs. 89.5% of cases treated with placebo. When comparing the counts of the different leukocyte subpopulations between both groups, it was observed that severe and critical patients treated with P2Et have higher total leukocyte counts (*p*: 0.0014) and absolute neutrophil counts (*p*: 0.013) with a tendency to present higher percentages of neutrophils (*p*: 0.065) (Table 4). In addition, they have lower percentages of eosinophils (*p*: 0.008), with a tendency to have lower absolute eosinophil counts (*p*: 0.040). In other cell populations and in the moderate ill COVID19 patients, no significant differences were found between the P2Et and the placebo group (Table 4).

**Table 4:** Differences between cell populations in the two arms and analyzed in moderate vs. severe or critical patients.

**Cell populatio*n***	**Group**	**All COVID-19 patients**				**Moderate COVID-19 patients**				**Severe and critical COVID-19 patients**			
		***n***	**Mean**	**SD**	***p*** **value**	***n***	**Mean**	**SD**	***p*** **value**	***n***	**Mean**	**SD**	***p*** **value**
Leukocytes /mm^3^	P2Et	36	7977.9	3103.9	0.060	25	7207.4	2963.3	0.427	11	9729.1	2790.9	**0.014**
	Placebo	38	6764.8	2318.9		22	6588.4	2211.3		16	7007.5	2511.9	
Neutrophils /mm^3^	P2Et	36	5347.3	2915.5	0.053	25	4538.5	2475.7	0.411	11	7185.4	3112.6	**0.013**
	Placebo	38	4167.7	2217.8		22	3962.5	2.252.1		16	4449.9	2210.2	
Neutrophils (%)	P2Et	36	64.6	12.9	0.113	25	61.5	11.9	0.346	11	71.7	12.8	0.065
	Placebo	38	59.4	15.1		22	57.5	164		16	61.9	13.2	
Monocytes /mm^3^	P2Et	36	625.4	209.3	0.213	25	611.4	1969	0.416	11	657.1	242.2	0.333
	Placebo	38	557.1	254.1		22	559.1	2405		16	554.5	279.8	
Monocytes (%)	P2Et	36	8.3	2.6	0.459	25	8.9	23	0.525	11	7.1	2.9	0.435
	Placebo	38	8.9	3.6		22	9.4	39		16	8.1	3.3	
Eosinophils /mm^3^	P2Et	36	107.5	92.6	0.052	25	119.6	964	0.386	11	79.8	80.4	**0.040**
	Placebo	38	159.9	130.9		22	149.3	1351		16	174.4	127.7	
Eosinophils (%)	P2Et	36	1.5	1.4	**0.017**	25	1.7	15	0.268	11	0.9	0.9	**0.008**
	Placebo	38	2.4	1.7		22	2.3	17		16	2.5	1.7	
Basophils /mm^3^	P2Et	36	45.0	34.4	0.677	25	40.2	214	0.406	11	55.7	53.5	0.922
	Placebo	38	48.9	46.2		22	45.8	237		16	53.3	66.6	
Basophils (%)	P2Et	36	0.6	0.4	0.219	25	0.6	04	0.186	11	0.6	0.5	0.606
	Placebo	38	0.7	0.6		22	0.7	04		16	0.7	0.8	
Lymphocytes /mm^3^	P2Et	36	1854.7	899.0	0.799	25	1900.3	908.5	0.833	11	1751.2	911.6	0.990
	Placebo	38	1804.4	796.6		22	1846.2	829.8		16	1747.0	771.4	
Lymphocytes (%)	P2Et	36	250	108	0207	25	274	103	0450	11	19.6	10.4	0.126
	Placebo	38	285	122		22	300	130		16	26.3	11.1	
Lymphocytes CD3 /mm^3^	P2Et	36	1226.4	631.0	0.659	25	1268.0	667.2	0.733	11	11318	557.7	0.919
	Placebo	38	1163.7	587.2		22	1203.2	619.8		16	11094	554.1	
Lymphocytes CD3 (%)	P2Et	36	66.3	11.1	0.453	25	66.0	11.4	0.770	11	66.9	10.9	0.437
	Placebo	38	64.3	11.6		22	65.0	11.3		16	63.2	12.2	
Lymphocytes CD4 /mm^3^	P2Et	36	688.2	411.0	0.637	25	691.1	425.2	0.640	11	681.7	396.7	0.880
	Placebo	38	646.5	345.9		22	636.7	357.3		16	659.9	340.8	
Lymphocytes CD4 (%)	P2Et	36	36.8	11.1	0.430	25	36.2	11.1	0.356	11	37.9	11.7	0.805
	Placebo	38	34.8	10.3		22	33.2	11.3		16	37.0	8.6	
Lymphocytes CD8 /mm^3^	P2Et	36	462.5	291.3	0.821	25	488.5	314.8	0.784	11	403.5	231.5	0.784
	Placebo	38	477.6	282.0		22	514.1	322.3		16	427.5	214.8	
Lymphocytes CD8 (%)	P2Et	36	25.7	10.0	0.790	25	25.6	8.6	0.388	11	26.1	13.2	0.627
	Placebo	38	26.3	8.7		22	27.9	9.5		16	24.2	7.2	
CD4+ CD8+ ratio	P2Et	36	1.7	0.9	0.404	25	1.6	0.9	0.580	11	1.8	1.1	0.430
	Placebo	38	1.5	0.7		22	1.5	0.9		16	1.6	0.6	
Lymphocytes CD4+ CD8+ /mm^3^	P2Et	36	29.0	81.0	0.249	25	35.4	96.6	0.313	11	14.4	15.9	0.647
	Placebo	37	13.3	14.1		22	14.1	16.8		15	12.1	9.2	
Lymphocytes CD4 + CD8 + (%)	P2Et	36	1.4	3.4	0.255	25	1.6	4.1	0.318	11	0.8	0.9	0.665
	Placebo	37	0.7	0.6		22	0.7	0.7		15	0.7	0.5	
Lymphocytes CD4-CD8- /mm^3^	P2Et	36	15.9	18.6	0.056	25	16.3	20.2	**0.041**	11	14.8	15.2	0.696
	Placebo	37	9.3	8.6		22	6.9	5.9		15	12.7	10.9	
Lymphocytes CD4-CD8- (%)	P2Et	36	0.9	1.1	**0.032**	25	0.9	1.0	**0.033**	11	1.0	1.2	0.378
	Placebo	37	0.5	0.4		22	0.4	0.3		15	0.7	0.5	

The bold values indicates *p* statistical significance (*p* < 0.05).

In the *T* cell population, P2Et seems to exert a protective role in COVID19 through the increase of the delta of CD3^+^CD4^−^CD8^−^ TL in severe illness patient (Supplementary Table 3) and the frequency and relative values of CD3^+^CD4^−^CD8^−^ cells in the treated group but with a preferential impact in the moderate COVID19 patients (Table 4). This effect is particularly evident in female population (Data no shown). Differences between moderate and severe patients in the *T* cell population between the placebo and P2Et groups can be found in the supplementary material (Supplementary Tables 2, 3).

### Patients treated with P2Et have an increase in innate cells in peripheral blood

**Figure 2:**
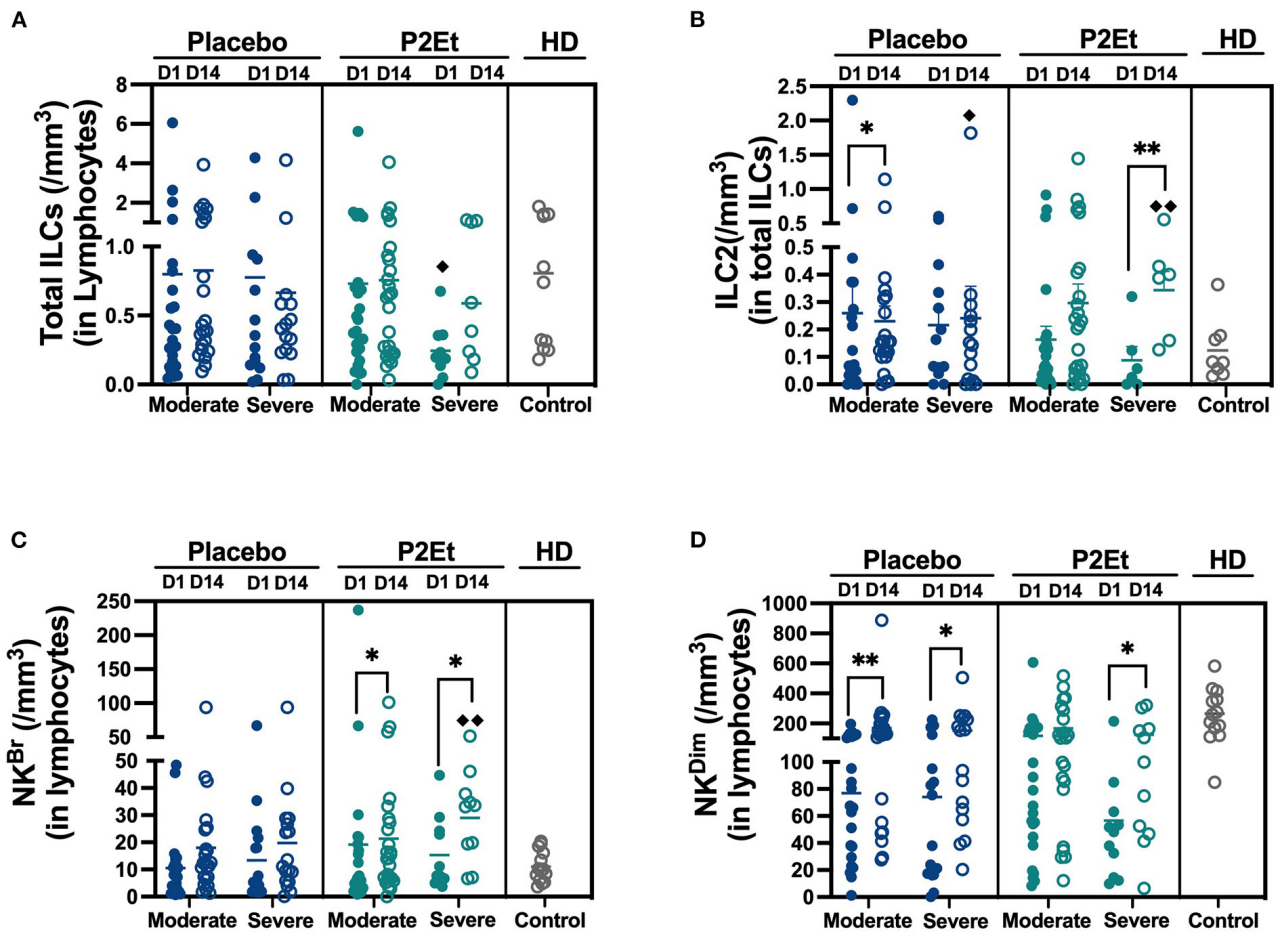
Absolute counts of Innate lymphocytes cells in the peripheral blood of COVID-19 patients. **(A)** Absolute counts of total Innate lymphocytes cells (ILCs) in COVID-19 patients (moderate and severe) treated with placebo or P2Et. Healthy donors (HD) were included. **(B)** Absolute counts of Innate type 2 lymphocytes cells (ILC2s) in COVID-19 patients (moderate and severe) treated with placebo or P2Et. HD were included. **(C)** Absolute counts of Natural killer Bright cells (NKBr) in COVID-19 patients (moderate and severe) treated with placebo or P2Et. HD were included. **(D)** Absolute counts of Natural killer Dim cells (NKDim) in COVID-19 patients (moderate and severe) treated with placebo or P2Et. HD were included. Data are represented as the mean ± SEM. The p values between Placebo and P2Et group or within groups were calculated using a Mann-Whitney test. ^*^p < 0.05, ^**^p < 0.01. The *p* values between COVID-19 patients and HD were calculated using a Mann-Whitney test. ♦*p* < 0.05, ♦♦*p* < 0.01.

A total of 40 patients either with moderate (*n* = 25) or severe (*n* = 15) disease in the placebo group or 37 patients either with moderate (*n* = 25) or severe (*n* = 12) disease in the P2Et group and 10 healthy donors (HD) were included in the analysis of innate lymphoid cells (ILCs) from peripheral blood. For the identification and analysis of ILCs, we used 15-parameter flow cytometry. The gating strategy is shown in Supplementary Figure 2. We did not find differences in Total ILCs numbers between the placebo and the P2Et group, however, we found a significant decrease in Total ILCs counts in severe patients from the P2Et group before treatment in comparison with HD (Figure 2A). After 14 days of treatment, we found a significant decrease in the ILC2 subset in moderate patients of the placebo group and a significant recovery of peripheral ILC2 in severe patients. Similarly, we observed a significant increase in the ILC2 subset in the severe group treated with P2Et as compared to the severe group of placebo and HD (Figure 2B).

Regarding the ILCp, we found that in the P2Et group there are no differences in response to treatment in both groups, contrary to the placebo group where a slight increase is observed. (Supplementary Figures 3A,B). The counts of ILC-1 remained unchanged (Supplementary Figures 3A,B and Supplementary Table 3).

Regarding NK cells, after 14 days of treatment we found a significantly recovery of NK^Br^ cells in P2Et treated group (moderate and severe), and even reaching higher levels than healthy individuals, suggesting the establishment of a better peripheral anti-viral immune response (Figure 2C). Although a recovery of NK^Dim^ was observed in moderate and severe placebo group, a better recovery of these population was evidenced in severe P2Et treated patients (Figure 2D). Other cell populations and activation markers were evaluated without differences between the groups (Supplementary Table 4).

### Modulation of inflammatory response in COVID-19 patients after P2Et treatment

**Figure 3:**
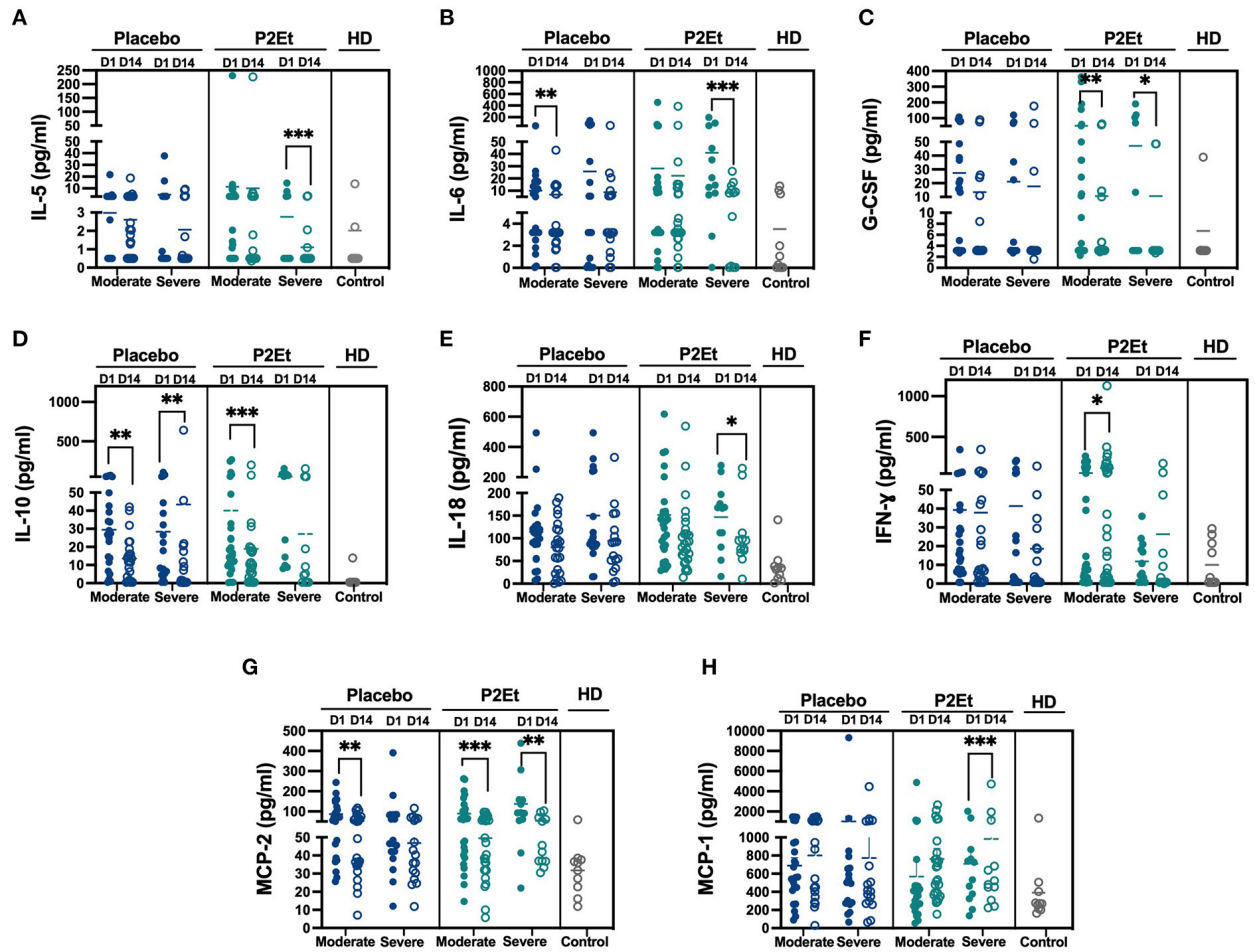
P2Et treatment modulate of inflammatory response in COVID-19 patients. Samples from placebo, P2Et and Healthy donors (HD) groups were analyzed by Milliplex to identify the quantity of cytokines present in the serum. **(A)** IL-5, **(B)** IL-6, **(C)** G.CSF, **(D)** IL-10, **(E)**. IL-18, **(F)** IFN-γ, **(G)** MCP-2, and **(H)** MCP-1 cytokines. Each dot represent one human sample. The *p* values between placebo and P2Et group or within groups were calculated using a Wilconxon test. ^*^p < 0.05, ^**^p < 0.01, ^***^p < 0.001.

A total of 40 patients with either moderate (*n* = 23) or severe (*n* = 17) disease in the placebo group or 39 patients with either moderate (*n* = 27) or severe (*n* = 12) disease in the P2Et group and 10 healthy donors (HD) were included in the analysis of 24 cytokines in the serum by Milliplex. We observed after 14 days of P2Et treatment a significantly decrease in IL-5, G-CSF and IL-18 (Figures 3A,C,E), in severe and/or moderate patients as indicated in the figure; as well as a mild increase in IFN-γ and MCP-1 (Figures 3F,H) which were not observed in the placebo group. In addition, IL-6 is reduced in moderate and severe patients in placebo, but more so in severe patients treated with P2Et (Figure 3B), and IL-10 is reduced in both groups but more so in moderate patients treated with P2Et (Figure 3D). Although MCP2 is reduced in moderate patients in the placebo group, a significant reduction is observed both in moderate and severe patients treated with P2Et (Figure 3G). We found no differences in the other cytokines evaluated such as: TNF-α, IL-2, IL-4, IL-7, IL-8, IL1-RA, IL-9, IL-13, IL-14, IL-15, IL-17, IL-18, IL-22, IP-10, MIP-α, TSLP, NT-PRO-BNP, between the placebo and P2Et group.

### P2Et exhibits antiviral activity against SARS-CoV-2

**Figure 4:**
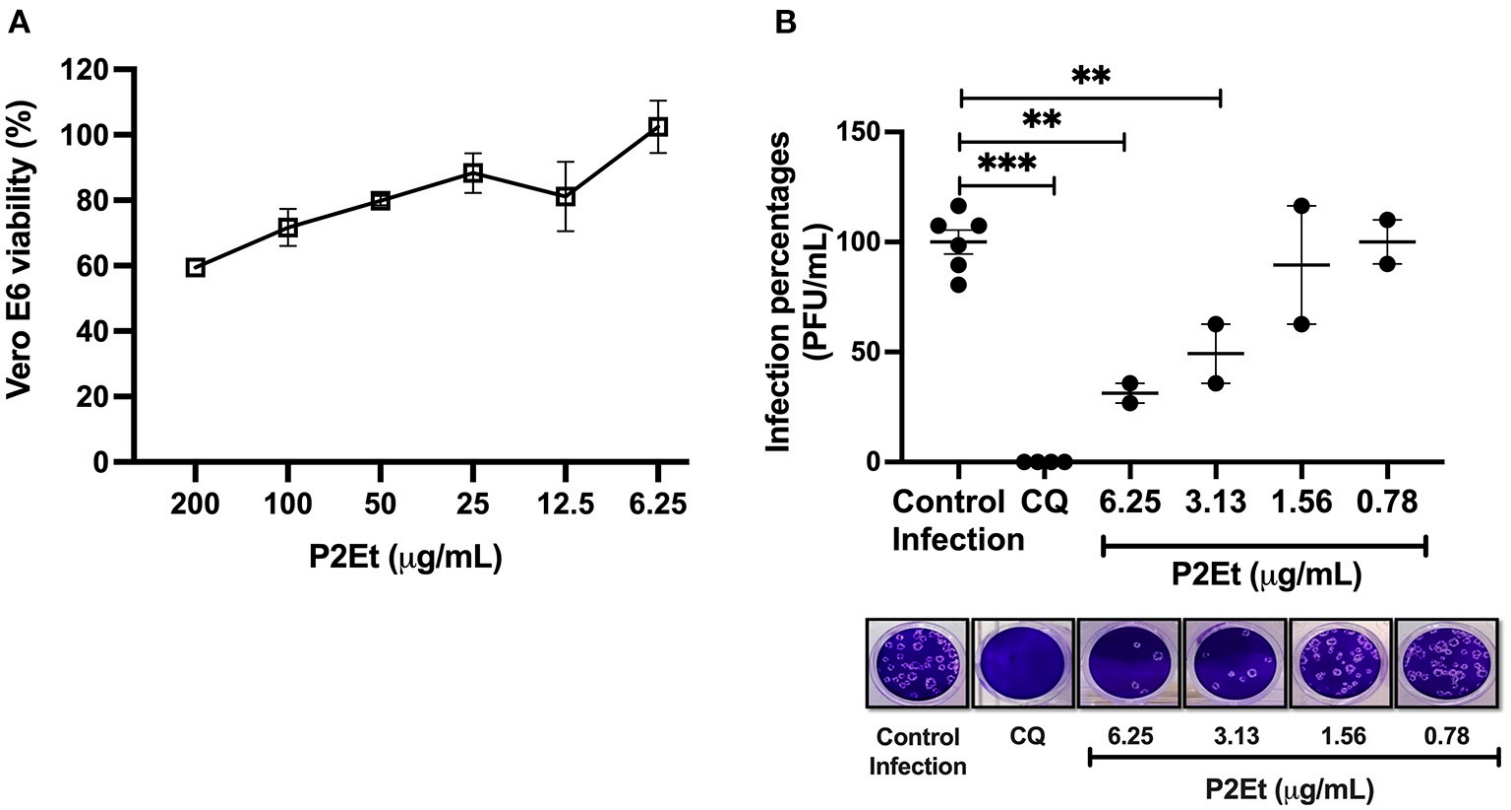
P2Et exhibit antiviral activity against SARS-CoV-2 with a concentration-depending effect. **(A)** Vero E6 viability after 48 h of P2Et treatment was evaluated by MTT assay (6.25-−200 μg/mL). Data were presented as Mean ± SEM. The viability percentages of the treated cell were calculated based on untreated control. Two independent experiments with three replicates each were performed (*n* = 6). **(B)** The SARS-CoV-2 titer (PFU/mL) was reduced in Vero E6 supernatants after pre-post treatment with P2Et (*n* = 2). CQ (100 μM) was used as a positive control of viral inhibition. Data are represented as the mean ± SEM. The *p* values were calculated using a Mann-Whitney test, ^**^p < 0.01, ^***^p < 0.001. Representative plaques of each treatment condition are shown.

Taking into account the decrease of the inflammatory response of the patients after treatment with P2Et, we evaluate if this could be due to an effect on the SARS-CoV-2 directly. For this, Vero E6 cells infected with SARS-CoV-2 were used. Vero E6 cells showed viability percentages higher than 90% when they were treated at 12.5 μg/mL or lower P2Et concentrations (Figure 4A). The CC50 value calculated for P2Et was 359.1 μg/mL. Cell viability was not affected by Cloroquine (CQ) treatment (positive control of viral inhibition) at 100 μM (27).

SARS-CoV-2 titers of 1.75 × 10^5^ PFU/mL and 2.75 × 10^5^ PFU/mL were obtained after P2Et treatment at 6.25 and 3.13 μg/mL, indicating inhibition percentages of 68.7% (*p*: 0.04) and 50.7% (*p*: 0.04), respectively, compared with the infection control (5.58 × 10^5^ PFU/mL). This antiviral effect was not observed at 1.56 and 0.78 μg/mL of P2Et (Figure 4B). The P2Et EC50 was 3.6 μg/mL, with a SI of 99.8. CQ treatment (positive control of viral inhibition) showed antiviral activity against SARS-CoV-2 at 100 μM (100 % inhibition, *p* < 0.0001) in Vero E6 cells (Figure 4B). Thus, we observed that P2Et showed antiviral activity against SARS-CoV-2 in a concentration-dependent manner.

### P2Et treatment reduces inflammation and fibrotic markers *in vivo* in a bleomycin model

**Figure 5:**
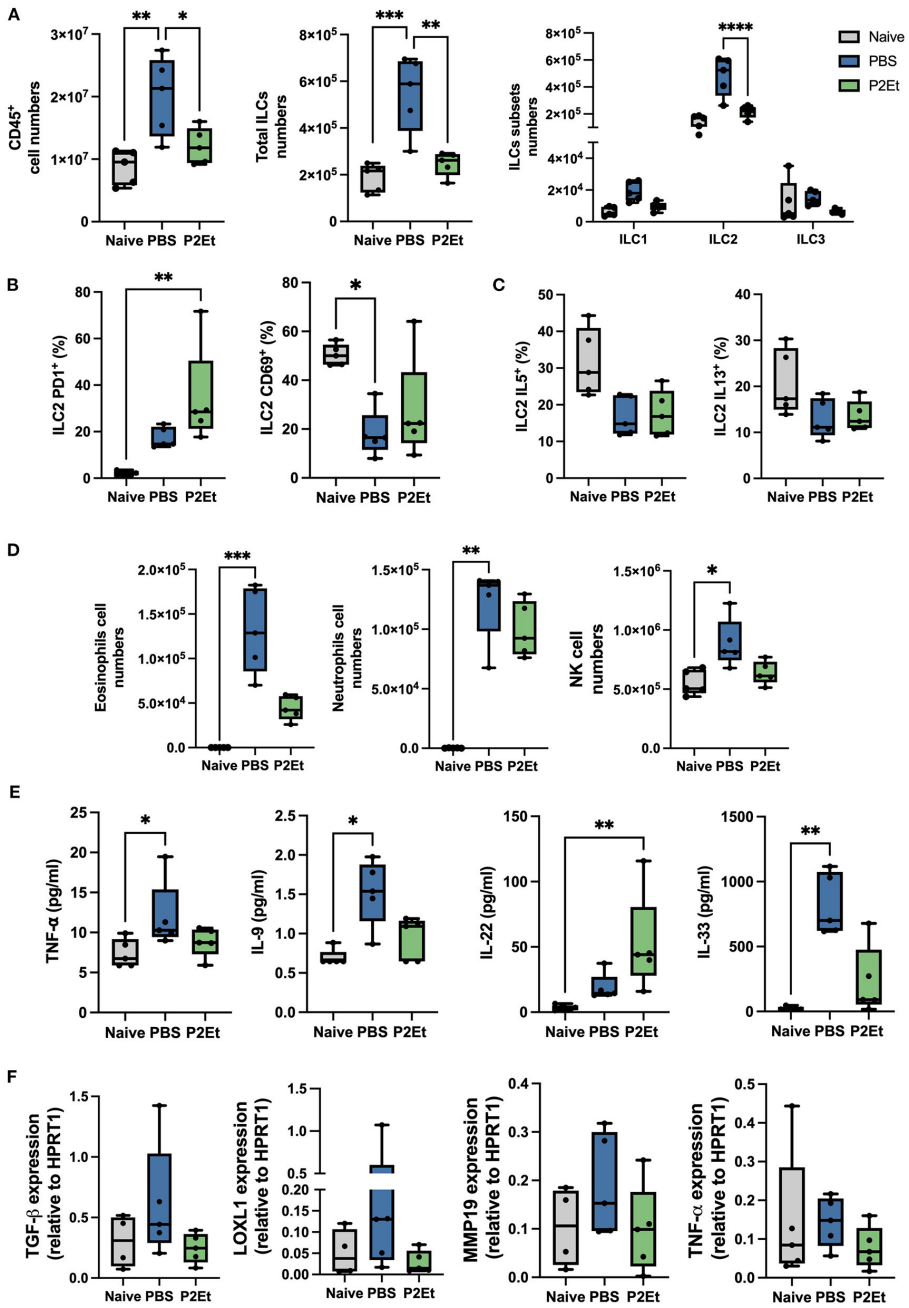
P2Et treatment reduces ILC2 the pro-fibrotic factors in the lung of bleomycine challenged mice. **(A)** Lungs from naïve animals were used as negative controls compared and to bleomycine challenged animals treated either with PBS or P2Et. Absolut counts from lung ex vivo samples are shown. **(B)** Frequency of PD1 and CD69 positive cells among the ILC2 subset is presented. **(C)** Part of the lung cell suspension was used for an overnight re-stimulation with IL-33 and IL-25 *in vitro*. Intracellular cytokine staining was performed, and the frequencies of IL-5 and IL-13 positive ILC2 are shown. **(D)** From the ex-vivo lung cell suspension eosinophils and neutrophils were identified, and absolute numbers are represented. **(E)** Samples from all groups were analyzed by LegendPlex to identify the quantity of cytokines present in the serum of each animal. **(F)** Lung tissue was analyzed by qPCR to check the mRNA expression of pro-fibrotic factors. All graphs are presented as box and whiskers graphs showing the min and max. The middle line is plotted at the median. Each dot represent one mouse. The *p* values were calculated using a Kruskall-Wallis test, ^*^p < 0.05, ^**^ *p* ≤ 0.01, ^***^p < 0.001, ^****^p < 0.001.

In this study the *in vivo* bleomycin model was used to evaluate the impact of the P2Et treatment *in situ* in lung inflammation and fibrotic markers expression. We observed that Bleomycin treatment significantly increased the number of CD45^+^ cells in the lungs compared to healthy controls. On the contrary, we did not observe this increase in the group treated with P2Et (Figure 5A). Furthermore, in the lungs we also observed that total ILCs were increased and that an increase on the total numbers of ILC2 was responsible for this observation in the animals treated with PBS (Figure 5A). We then assessed the activation state and the functionality of lung ILC2s and we found no differences in the expression of the activation marker CD69 among our PBS treated group and the P2Et treated animals, but interestingly, we observed a significant increase in the expression of PD1 at the surface of the ILC2 only in the P2Et group (Figure 5B). We observed that ILC2s from Bleomycin challenged mice might produce less IL-5 and IL-13 in frequency than ILC2s from healthy animals but no difference was found between the two challenged groups (Figure 5C). However, since the number of ILC2 observed in the Bleomycin challenged PBS treated mice was significantly higher than the P2Et group, we assessed the impact in the eosinophil and neutrophil numbers in the lungs. Eosinophils and neutrophils in the lungs of naïve animals were rare contrary to the bleomycin challenged group were these two-populations increased significantly, especially in the PBS group. Animals treated with P2Et showed much less recruitment of eosinophils than the control group, but not for neutrophils (Figure 5D). In addition, we observed that NK cells increased in a significant manner in the lungs of control mice while the numbers were much lower in the treated group (Figure 5D). In the serum some specific markers of fibrosis development were evaluated. We observed that 2 fibrogenic factors were significantly increased in the PBS group, TNFα and IL-9 (Figure 5E). In contrast, their increase was not significant in the P2Et group. Moreover, we observed IL-33 in the serum of the PBS group and only the group treated with P2Et showed an increased in IL-22 serum levels.

To finish, we assessed TGF-β, LOXL1, MMP19, and TNF- α directly in lung tissue by qPCR. We observed a tendency of increase of all these factors in the PBS group but not in the P2Et where their upregulation was controlled (Figure 5F). All together these results suggest that P2Et can control several pro-fibrotic factors what could be translated by a less severe disease and less secondary effects after the initial acute infection.

## Discussion

Patients with severe COVID-19 not only develop pulmonary disease, but it eventually end up in acute respiratory distress syndrome (ARDS), Acute Lung Injury (ALI), and displaying a myriad of extrapulmonary symptoms, including acute kidney injury, acute cardiac injury, coagulopathy, thromboembolic complications, such as stroke and pulmonary embolism, and circulatory shock, caused by severely persistent inflammation. The detection of SARS-CoV-2 in multiple organs, the presence of thrombosis and ischemic complications, and its multisystemic clinical features, suggest that COVID-19 might be a systemic vascular disease with inflammation and coagulation localized within the lungs (29). Thus, antiviral therapy and anti-inflammatory agents are currently the best options for the treatment of COVID-19 patients (30).

Several available vaccines have been shown to be effective against serious COVID-19 infection across all ages, preventing severe disease, hospitalization, and death. Reports from the Public Heath England showed that some of them as Pfizer-BioNTech and Oxford-AstraZeneca vaccines are highly effective in reducing symptomatic infections even among older people. However, vaccination does not show 100% of efficacy and breakthrough infections can occur even in vaccinated individuals (31), pointing the importance of continuing the search for alternative COVID-19 treatments.

The results obtained in this double-blind controlled clinical study support previous evidence on the use of polyphenolic compounds in the treatment of respiratory diseases. Plant derived polyphenols are endowed with antioxidant and anti-inflammatory, immune, antitumor, and prebiotic properties, giving them useful properties for the treatment of a wide array of chronic and viral diseases. The effectiveness of polyphenols has been sustained by some of these biological functions, but also by their powerful antiviral activities, reflected by the modulation of viral proteins and host receptors, and even by the restoration of microbiota that influence the immune response against virus (32).

During this clinical trial we observed that patients treated with the phytomedicine were discharged from the hospital 2.7 days before the placebo group; this represents lower costs for the local health system, particularly in hospitalization, resulting in more beds available for new patients. All patients in the study showed a reduction in the inflammatory markers at the time of hospital discharged; we identified some statistically significant differences in the phytomedicine treated patients, related with the homeostasis of some hematopoietic and immune cells such as, (i) recovery of total ILCs, specially of the ILC2 subpopulation in peripheral blood of most severe patients; (ii) increase in the NK^dim^ population also in severe patients; (iii) increase of NK^bright^ in both moderate and severe patients; (iv) increase of the expression of the ICOS activation marker (v) reduction of peripheral eosinophils; (vi) increasing of CD4^−^CD8^−^ *T* cells, and (vii) increase in CD4^+^CD8^+^ T cells in moderate illness patients.

Innate lymphoid cells as NK and helper ILCs are innate lymphocytes that can be found in peripheral blood but also in peripheral tissues. They respond fast and strongly without prior sensitization and are important in tumor surveillance, immune homeostasis and as early cytotoxic (NK) cells during viral infections. It has been reported that in acute SARS-CoV-2 infection, both NK^bright^, and NK^dim^ cell subset drop in cell numbers in circulation, and this occurs even during mild infection (33). This drop of circulating NK cell reflects active homing of the NK to the lungs since increase NK frequencies have been observed in bronchioalveolar lavage (BAL) in patients (34, 35). NK cell hyperactivation, likely driven by IL-6, IL6R and IL-18 is a feature of severe COVID-19 as compared to mild or moderate disease. These results are more significant in the long-term recovery of COVID patients. The increased production of IL-6 and particularly of IL-18, supposes the activation of the NLRP3 inflammasome, which may be related to the intracellular recognition of the virus by pattern recognition receptors such as Toll-like receptors 3, 7, 8, and 9 and viral-infection sensors RIG-I and MDA5 (36). This increase could be related to pyroptotic cell death and possibly inflammation and coagulopathy. It would be important to evaluate if treatment with P2Et reduces the activation of the inflammasome, which would be relevant in the control of symptoms, not only respiratory but also vascular. The reduction of IL-18 observed after treatment with P2Et, could be simply related to a reduction in viral load.

Extensive literature supports a clear correlation between respiratory viral infections and the development of fibrosis (37). In SARS-CoV2 is now know that lung fibrosis is one of the main problems even after the recovery from the acute phase of the infection but the molecular basis are still unclear and are believed to be multifactorial (37). In our murine bleomycin fibrosis model, we showed a high frequency of innate immune cells. Interestingly, we showed first a statistically significant reduction of CD45^+^ cells in lungs of P2Et treated mice, suggesting that the treatment might have a protective role against local inflammation. In addition, we also showed a significant reduction of ILC2 in tissue with higher expression of PD1 and a limited NK infiltration in lungs of treated animals. ILC2 are known to be involve in lung tissue damage after fibrosis induction and PD1 has been reported as a negative regulator of ILC2 function in both human and mice (38) suggesting an impairment after the treatment with P2Et. It is possible that after treatment these cells leave the lungs to get back into the circulation and then increase in peripheral blood as it was shown for NK in COVID-19 patients. When activated by tissue derived alarmins as IL-33, ILC2 produces important amounts of type 2 cytokines as IL-5 and IL-13. This cytokine is one of the most important ones for eosinophils maturation and differentiation in mice and humans (39). In our case, the murine model showed that animals without treatment significantly increases the number of eosinophils found in the lungs what could be a consequence of the higher expression of IL-33 that will activate and recruit higher numbers of IL-5-secreting-ILC2s. On the contrary, we observe that treated animals have lower IL-33 and IL-9 serum levels, lower ILC2 and eosinophils numbers what reenforces the systemic anti-inflammatory role of the P2Et. In fact, eosinophils express biologically active IL-9 that is linked to the pathogenicity of airway allergic diseases as asthma by influencing the recruitment of effectors cells (40).

Accordingly, during the clinical study we showed that patients treated with P2Et have lower numbers of eosinophils in peripheral blood compared to the placebo group, especially women. The decreased of these cells in the patients could also be associated to the lower amounts of G-CSF, IL-5, IL-6, IL-13, IL-17, and TGF-β observed in the treated group compared to the placebo group. Although in our study we did not observe a direct correlation between the decrease in eosinophils and any of these cytokines, we cannot rule out that they may be influenced by this decrease, some of these cytokines are known to be involved in eosinophil differentiation and all can be produced by these types of cells. It can also be considered that the decrease in the cell numbers and the circulating factors might be associated with less tissue damage and finally with better prognosis (41). In line with these results, our animal model showed that a profibrotic treatment as the bleomycin, significantly increase the serum levels of TNF-α and IL-9 and that in contrast, the increase of these two factors was not significant after the P2Et treatment. In addition, IL-22 has been reported as an inhibitory factor of fibrosis and it was also found in significant quantities only in the treated group, suggesting that the P2Et could also have a role in controlling systemic signs of the fibrotic process.

Another interesting finding was the significant increase in double negative (DN) LTs in response to P2Et treatment. Recently, an increase in DN cells have been evidenced in COVID-19 patients (42), however, their role in the pathogenesis of the disease is not clear. Although we did not establish what type of DN LT are expanded (43), the results suggest that they could have a protective role in COVID-19 patients as previously reported in influenza (44). A future challenge is to evaluate whether the polyphenols present in P2Et exert their immunomodulatory role through the modulation of this population. Considering the set of results, we can hypothesize that P2Et treatment decreases lung inflammation by the modulation of the IL-33-ILC2-Eosino-IL-9 axis in early timepoints, but also trough the effect on innate immune populations. In fact, it has been shown that some polyphenols are able to modulate inflammatory responses and migratory mechanisms on immune cells (45, 46).

**Figure 6:**
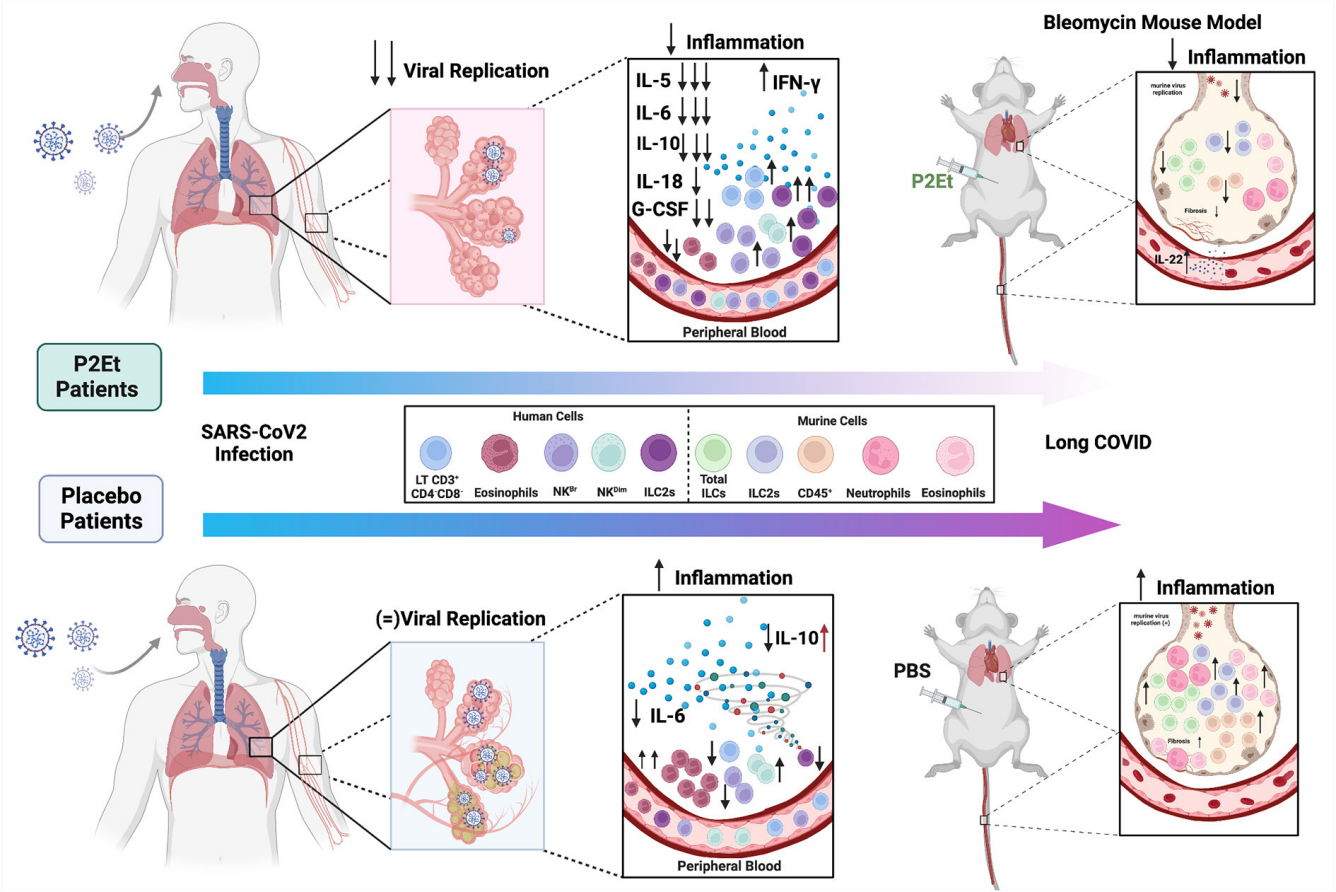
Proposed model for the P2Et effect in COVID-19. COVID-19 patients (moderate or severe) were treated with P2Et or Placebo. P2Et treatment (upper left) decreases ex vivo viral replication as well as proinflammatory cytokines such as IL-5, IL-6, IL-10, IL-18, G-CSF, and increases IFN-γ in peripheral blood. A decrease in eosinophils and an increase in other cell populations such as LT CD3+CD4-CD8-, NK^Br^, NK^Dim^, and ILC2s are observed. The upper right image shows the effect of P2Et treatment in a mouse model of bleomycin-induced pulmonary fibrosis. In the lung, it decreases CD45+ cells, total ILC, ILC-2, eosinophils, and neutrophils. In peripheral blood it increases the production of IL-22. In summary, P2Et treatment regulates the inflammation induced by SARS-CoV-2 infection, modulating factors related to long-term clinical manifestations. The lower left image shows the effect of Placebo treatment in patients with COVID-19. A slight decrease in IL-6 and IL-10 is observed in moderate patients but an increase in IL-10 in severe patients. For the other cytokines (IL-5, IL-18, G-CSF, and IFN-γ) no significant changes are observed. On the contrary, in this group of patients, an increase in eosinophils and a slight increase in NK^Dim^ cells, as well as a decrease in LT CD3^+^CD4^−^CD8^−^, NK^Br^ and ILC2s, are observed. In the PBS mouse model (negative control) there is evidence of an increase in CD45^+^ cells, total ILCs, ILC-2s, eosinophils, and neutrophils as well as a slight increase in markers related to pulmonary fibrosis in the lung that show the exaggerated inflammation. Image created using BioRender (https://Biorender.com/).

Taking all of the above into account, we propose that P2Et regulates the inflammation induced by SARS-CoV-2 infection, modulating factors related to long-term clinical manifestations, possibly related to its antiviral capacity, but also due to the decrease in innate response in the lung which is related to lung damage (Figure 6). P2Et should be used as soon as it is diagnosed to prevent the disease from getting worse. Despite the large amount of biological evidence, the challenge for the development of good plant extracts that can comply with the route of development of polymolecular drugs have been related to agricultural, pharmacotechnical, bioavailability and stability factors. The results of this study provide motivating elements in the discovery of polymolecular drugs, which, in addition to having a non-negligible traditional use, have lower toxicity given their molecular diversity and the possibility of acting synergistically on various molecular targets.

## Data availability statement

The original contributions presented in the study are included in the article/Supplementary materials, further inquiries can be directed to the corresponding author.

## Ethics statement

The studies involving human participants were reviewed and approved by the independent Ethics Committees (IECs) of the Hospital Universitario San Ignacio (Approval: 2020/050) and Centro Cardiovascular Colombiano Clínica Santa María (Approval: 2021/177). Trial was registered with the number No. NCT04410510 (https://clinicaltrials.gov/ct2/show/NCT04410510). The patients/participants provided their written informed consent to participate in this study. The animal study was reviewed and approved by Veterinary Authority of the Swiss Canton Genève (authorization no. GE119/20).

## Author contributions

CU, RB-R, AB, and SF were responsible for the study design, interpretation of results, drafting and revising the manuscript. AG, CM, and PA participated in the recruitment of the COVID19 patients, acquisition, and analysis of clinical safety and efficacy data. KP, MZ-C, LF-A, WZ-B, and MR were responsible for the design, acquisition, and analysis of the *in vitro* COVID19 model. AG-C, HE-A, and CJ were responsible of the design, acquisition, and analysis of the *in vivo* lung model. All authors participated in drafting and revising the manuscript and have read and approved the final manuscript.

## Funding

Funding was provided by the Ministerio de Ciencias (MINCIENCIAS) in the Mincienciaton grant (Contract 378-2020, Code 1203101576633). Additionally, RB-R and CU disclosed receipt of the following financial support for Administrative Department of Science, Technology and Innovation COLCIENCIAS (792-2017 2nd Call Scientific Ecosystem “Generation of therapeutic alternatives in cancer from plants through research and translational development, articulated in environmentally and economically sustainable value systems”), World Bank and Vicerrectoría de Investigaciones, Pontificia Universidad Javeriana, Bogotá, Colombia (contract no. FP44842-221-2018), MINCIENCIAS, Ministerio de Educación Nacional, Ministerio de Industria, Comercio y Turismo and ICETEX.

## Conflict of interest

Authors SF and CU are inventors of a granted patent related to P2Et. Authors SF, CU, and RB-R are partners of the DreemBio company who was a licensee of related patents. The remaining authors declare that the research was conducted in the absence of any commercial or financial relationships that could be construed as a potential conflict of interest.

## Publisher's note

All claims expressed in this article are solely those of the authors and do not necessarily represent those of their affiliated organizations, or those of the publisher, the editors and the reviewers. Any product that may be evaluated in this article, or claim that may be made by its manufacturer, is not guaranteed or endorsed by the publisher.
