# Supplementary material for: Randomized double-blind clinical study in patients with COVID-19 to evaluate the safety and efficacy of a phytomedicine (P2Et)
Source: Front Med (Lausanne). 2022 Sep 8;9:991873. doi: 10.3389/fmed.2022.991873 (PMC9494348; doi:10.3389/fmed.2022.991873)
Supplement: Supplementary file 1 [file Data_Sheet_1.pdf]

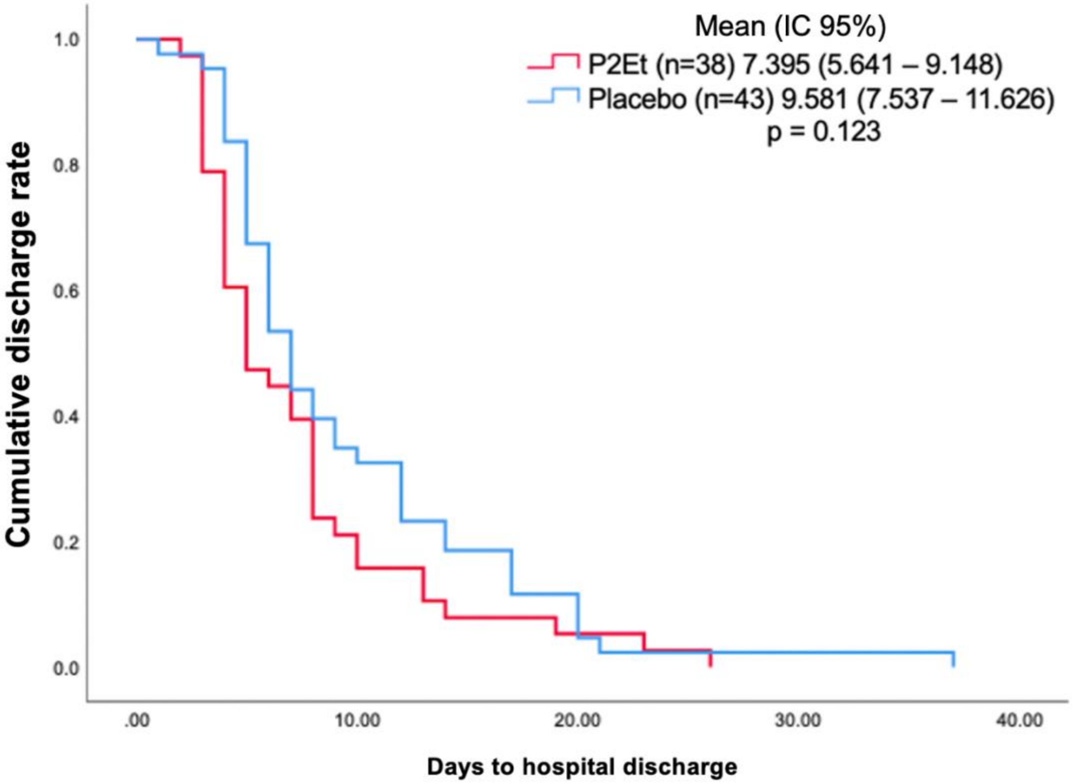

Supplementary Figure 1. Time to hospital discharge in patients with COVID19. The length of hospitalization was calculated from the first day in the hospital that was the same day of the inclusion and randomization, and the last day of hospitalization. The *p* value was calculated using Log-rank Test

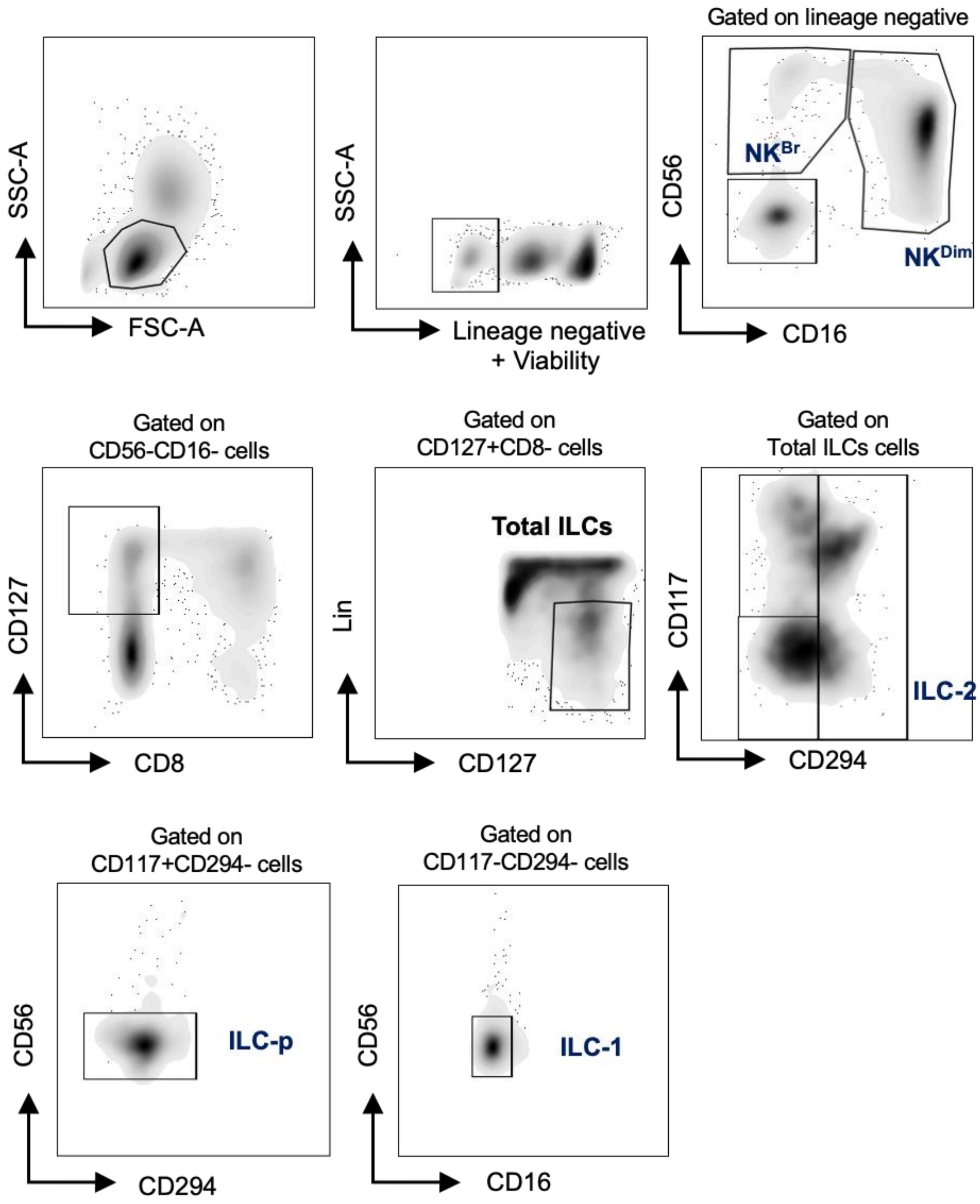

Supplementary Figure 2. Representative Flow Cytometry Analysis of ILCs cells in peripheral Blood. Innate lymphocytes cells (ILCs) were identified within the peripheral blood lymphocyte region on the basis of their forward (FSC-A) and side scatter (SSC-A) profiles (FSC low and SSC low) and by excluding from the analysis doublets (FSC-H/FSC-W dot plot, followed by SSC-A/SSC-W dot plot) and dead cells (positive for Zoombie green LIVE/DEAD fixable dead cell stain kit). NK^{Br} were gated as $CD56^{+}CD16^{-}$ and NK^{Dim} cells were gated as $CD56^{+}CD16^{+}$. Total ILCs were gated as $CD56^{-}CD16^{-}CD8^{-}Lin^{-}CD127^{+}$ cells. ILC-1 cells were gated on Total ILCs as $CD117^{-}CD294^{-}CD56^{-}CD16^{-}$, ILC-2 cells were gated on Total ILCs as $CD117^{+}CD294^{+}$ and ILC-p were gated on Total ILCs as $CD117^{+}CD294^{-}CD56^{-}$

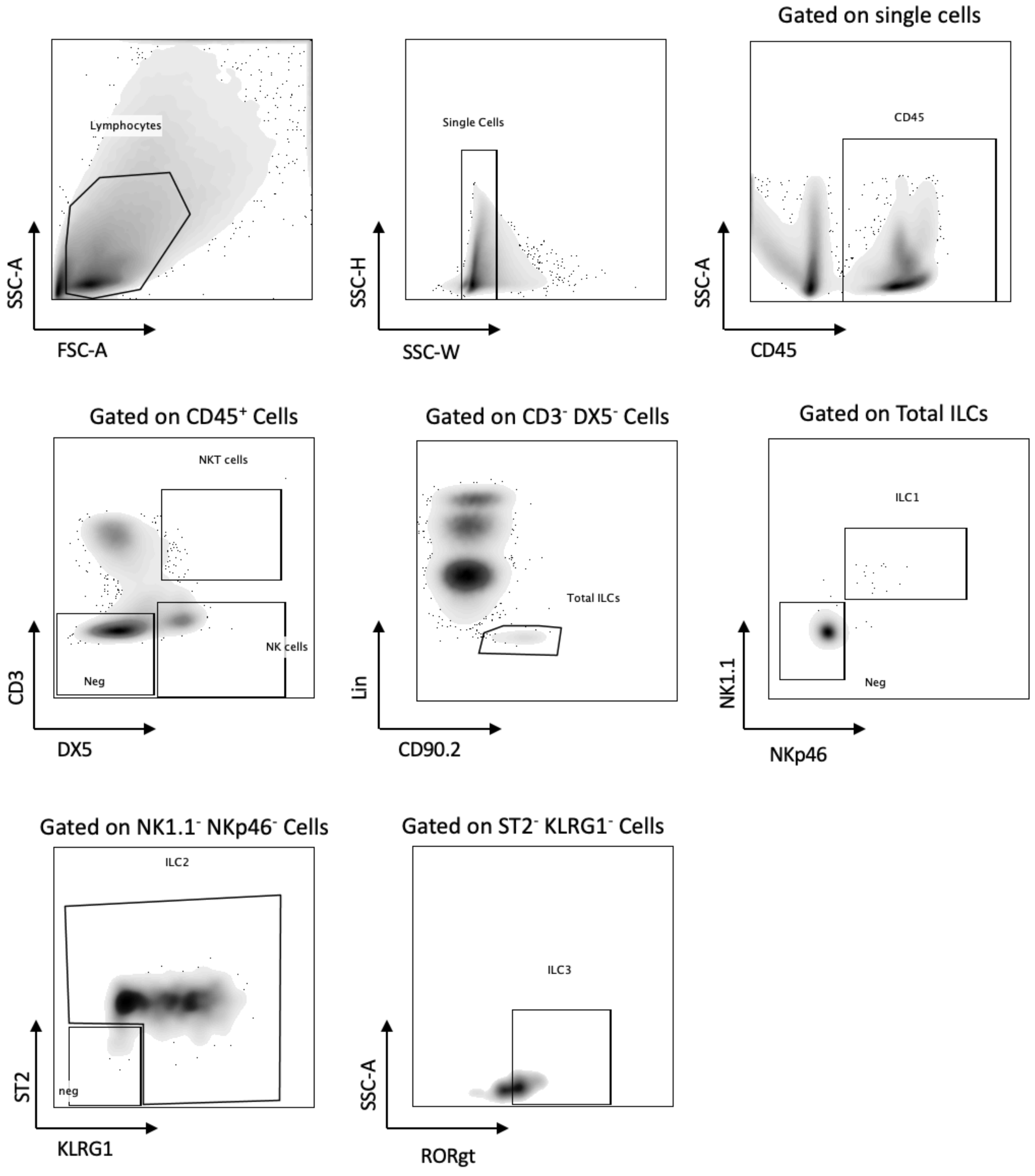

Supplementary Figure 3. Representative Flow Cytometry Analysis of ILCs cells in mouse lungs. Innate lymphocytes cells (ILCs) were identified in the tissue within the lymphocyte region on the basis of their forward (FSC-A) and side scatter (SSC-A) profiles after excluding doublets (SSC-H/SSC-W). NKs were gated as $CD45^{+}CD3^{-}$ $DX5^{+}$. Total ILCs were gated as Lin^{-} $CD90^{+}$ among the $CD45^{+}CD3^{-}$ $DX5^{-}$. ILC1s from the total ILC gate are consider $NK1.1^{+}$ $NKp46^{+.}$ From the negative population we identified ILC2 as $ST2^{+}$ $KLRG1^{+}$ and from that negative gate ILC3 are $RORgt^{+}$.

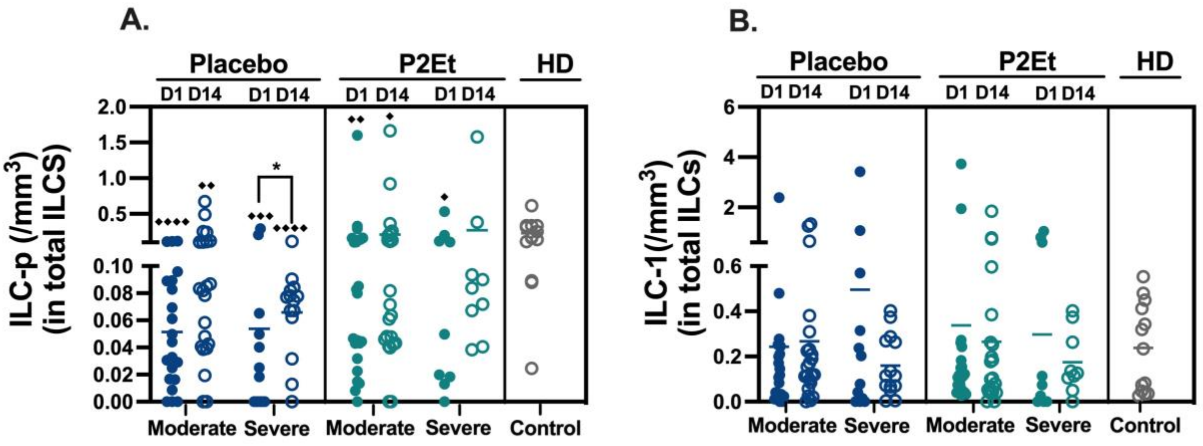

Supplementary Figure 4. Absolute Counts of ILCp and ILC-1 subpopulations in COVID-19 patients. A. Absolute counts of Innate lymphocytes precursors cells (ILCp) in COVID-19 patients (moderate and severe) treated with placebo or P2Et. Healthy donors (HD) were included B. Absolute counts of Innate type 1 lymphocytes cells (ILCs) in COVID-19 patients (moderate and severe) treated with placebo or P2Et. HD were included. Data are represented as the mean ± SEM. The p values between Placebo and P2Et group or within groups were calculated using a Mann-Whitney test. *$p < 0.05$, **$p < 0.01$. The p values between COVID-19 patients and HD were calculated using a Mann-Whitney test. ◆$p < 0.05$, ◆◆$p < 0.01$, ◆◆◆$p < 0.001$, ◆◆◆◆$p < 0.0001$.

Table S1. Summary of Adverse Events by Frequency presented in the P2Et group and placebo arm.

Description	Placebo					P2Et				Overall				
	Grade 1 (n=51)	Grade 2 (n=24)	Grade 3 (n=2)	Grade 4 (n=6)	Grade 5 (n=1)	Grade 1 (n=47)	Grade 2 (n=24)	Grade 4 (n=2)	Grade 5 (n=2)	Grade 1 (n=98)	Grade 2 (n=48)	Grade 3 (n=2)	Grade 4 (n=8)	Grade 5 (n=3)
Sinus bradycardia	2 (3.9%)	0 (0%)	0 (0%)	0 (0%)	0 (0%)	1 (2.1%)	0 (0%)	0 (0%)	0 (0%)	3 (3.1%)	0 (0%)	0 (0%)	0 (0%)	0 (0%)
Hypertriglyceridemia	7 (13.7%)	1 (4.2%)	0 (0%)	1 (16.7%)	0 (0%)	5 (10.6%)	0 (0%)	0 (0%)	0 (0%)	12 (12.2%)	1 (2.1%)	0 (0%)	1 (12.5%)	0 (0%)
Fever	3 (5.9%)	0 (0%)	0 (0%)	0 (0%)	0 (0%)	1 (2.1%)	1 (4.2%)	0 (0%)	0 (0%)	4 (4.1%)	1 (2.1%)	0 (0%)	0 (0%)	0 (0%)
hypokalemia	1 (2.0%)	1 (4.2%)	0 (0%)	0 (0%)	0 (0%)	2 (4.3%)	1 (4.2%)	0 (0%)	0 (0%)	3 (3.1%)	2 (4.2%)	0 (0%)	0 (0%)	0 (0%)
Skin rash	2 (3.9%)	0 (0%)	0 (0%)	0 (0%)	0 (0%)	1 (2.1%)	0 (0%)	0 (0%)	0 (0%)	3 (3.1%)	0 (0%)	0 (0%)	0 (0%)	0 (0%)
Abdominal pain	0 (0%)	1 (4.2%)	0 (0%)	0 (0%)	0 (0%)	1 (2.1%)	0 (0%)	1 (50.0%)	0 (0%)	1 (1.1%)	1 (2.1%)	0 (0%)	1 (12.5%)	0 (0%)
Symptomatic focal epilepsy	0 (0%)	1 (4.2%)	0 (0%)	0 (0%)	0 (0%)	0 (0%)	0 (0%)	0 (0%)	0 (0%)	0 (0%)	1 (2.1%)	0 (0%)	0 (0%)	0 (0%)
Subarachnoid hemorrhage right frontal lobe	0 (0%)	0 (0%)	0 (0%)	1 (16.7%)	0 (0%)	0 (0%)	0 (0%)	0 (0%)	0 (0%)	0 (0%)	0 (0%)	0 (0%)	1 (12.5%)	0 (0%)
Acute kidney injury	0 (0%)	0 (0%)	0 (0%)	1 (16.7%)	0 (0%)	0 (0%)	0 (0%)	0 (0%)	0 (0%)	0 (0%)	0 (0%)	0 (0%)	1 (12.5%)	0 (0%)
Acute and moderate respiratory distress syndrome	0 (0%)	0 (0%)	0 (0%)	2 (33.3%)	0 (0%)	0 (0%)	0 (0%)	1 (50.0%)	0 (0%)	0 (0%)	0 (0%)	0 (0%)	3 (37.5%)	0 (0%)
Severe acute respiratory distress syndrome	0 (0%)	0 (0%)	0 (0%)	1 (16.7%)	0 (0%)	0 (0%)	0 (0%)	0 (0%)	0 (0%)	0 (0%)	0 (0%)	0 (0%)	1 (12.5%)	0 (0%)
Severe viral pneumonia by SARS-CoV2	0 (0%)	0 (0%)	0 (0%)	0 (0%)	1 (100%)	0 (0%)	0 (0%)	0 (0%)	0 (0%)	0 (0%)	0 (0%)	0 (0%)	0 (0%)	1 (33.3%)
Headache	0 (0%)	0 (0%)	0 (0%)	0 (0%)	0 (0%)	3 (6.4%)	0 (0%)	0 (0%)	0 (0%)	3 (3.1%)	0 (0%)	0 (0%)	0 (0%)	0 (0%)
Septic shock of pulmonary origin	0 (0%)	0 (0%)	0 (0%)	0 (0%)	0 (0%)	0 (0%)	0 (0%)	0 (0%)	1 (50.0%)	0 (0%)	0 (0%)	0 (0%)	0 (0%)	1 (33.3%)
Cardiorespiratory failure	0 (0%)	0 (0%)	0 (0%)	0 (0%)	0 (0%)	0 (0%)	0 (0%)	0 (0%)	1 (50.0%)	0 (0%)	0 (0%)	0 (0%)	0 (0%)	1 (33.3%)
Others	36 (70.6%)	20 (83.3%)	2 (100%)	0 (0%)	0 (0%)	33 (70.2%)	22 (91.67%)	0 (0%)	0	70 (71.4%)	42 (87.5%)	2 (100%)	0 (0%)	0 (0%)

Table S2 Differences between T lymphocytes in the P2Et group vs placebo in patients with moderate illness

Moderate Illness Parameter		Placebo (n=21) D1	Placebo (n=21) D14	P2Et (n=23) D1	P2Et (n=23) D14	P value
CD3+	(mm^3)	626,0000	1237,7381	807,7435	1292,5957	ns
	%	62,0524	65,5190	59,6130	67,3957	ns
	Delta %	3,47		7,78		0,078
CD4+	(mm^3)	333,8952	659,2005	447,7348	697,2696	ns
	%	33,3333	33,8762	33,0913	36,7826	ns
	Delta %	0,54		3,69		0,252
CD8+	(mm^3)	260,7095	524,1143	308,3174	501,5739	ns
	%	25,6429	27,5524	22,5174	26,1913	ns
	Delta %	1,91		3,67		0,189
CD4 CD8 Ratio	(mm^3)	1,5081	1,5067	1,7709	1,6478	ns
	Delta %	0,00		-0,12		0,311
CD4+ CD8+	(mm^3)	9,0090	14,7429	13,9600	38,2900	ns
	%	0,8905	0,7619	1,2609	1,7565	ns
	Delta %	-0,13		0,50		**0,035***
CD4- CD8-	(mm^3)	5,3000	6,9005	11,0739	16,8991	ns
	%	0,5333	0,3762	0,7522	0,9261	ns
	Delta %	-0,16		0,17		0,066
Lymphocytes T Gamma Delta	(mm^3)	18,9624	33,5005	25,7022	37,6957	ns
	%	1,8190	1,9095	1,8870	1,9913	ns
	Delta %	0,09		0,10		0,453

Delta was calculated from the difference from percentage of day 14 minus percentage of day 1. The p value is only shown in the delta for each parameter, ns means p value > 0.05

Table S3. Differences between cell populations of T lymphocytes between the P2Et group and placebo in patients with severe illness

Severe and critical Illness Parameter		Placebo (n=12) D1	Placebo (n=12) D14	P2Et (n=10) D1	P2Et (n=10) D14	P value
CD3+	(mm^3)	501,9583	1035,0083	666,9900	1149,3100	ns
	%	62,0333	63,0833	58,5200	69,0200	ns
	Delta %	1,05		10,50		0,104
CD4+	(mm^3)	253,0500	577,0667	336,4900	699,9700	ns
	%	29,8750	34,6917	32,1700	39,3800	ns
	Delta %	4,82		7,21		0,500
CD8+	(mm^3)	210,9875	416,1150	297,3900	409,1320	ns
	%	27,5500	24,9750	23,9800	27,1200	ns
	Delta %	-2,58		3,14		0,104
CD4 CD8 Ratio	(mm^3)	1,3625	1,4658	1,6930	1,8650	ns
	Delta %	0,10		0,17		0,416
CD4+ CD8+	(mm^3)	8,7300	12,9817	23,8600	11,7860	ns
	%	1,2333	0,7833	1,5100	0,7100	ns
	Delta %	-0,45		-0,80		0,090
CD4- CD8-	(mm^3)	8,9492	9,0550	1,8710	10,7420	ns
	%	0,9583	0,5508	0,1900	0,8300	ns
	Delta %	-0,41		0,64		**0,015**
Lymphocytes T Gamma Delta	(mm^3)	19,4558	43,1667	7,8100	27,8100	ns
	%	2,3000	2,3750	0,7100	0,9800	ns
	Delta %	0,08		0,27		0,223

Delta was calculated from the difference of percentage of day 14 minus percentage of day 1. The p value is only shown in the delta for each parameter, ns means p value > 0.05

Table S4. Differences between innate cell populations in the P2Et and placebo group in patients with moderate and severe illness

		Moderate COVID19				Severe and critical COVID19			
Cell population (mm^3)	Groups	n	mean	SD	p value	n	mean	SD	p value
Total ILC	P2Et	26	0,7565	0,8095	0.679	7	0,6605	0,4093	0.447
	Placebo	22	0,8487	0,8876		15	0,6652	1,0099	
ILC-2	P2Et	26	0,2217	0,2349	0.820	6	0,3435	0,1665	***0.036****
	Placebo	22	0,2299	0,2617		15	0,2416	0,4497	
ILC-2 CD25+	P2Et	25	0,1430	0,1595	0.303	8	0,0604	0,0539	0.697
	Placebo	20	0,0791	0,0751		16	0,0670	0,0826	
ILC-2 CD27+	P2Et	25	0,0109	0,0222	0.077	6	0,0460	0,0613	0.203
	Placebo	20	0,0270	0,0542		16	0,0393	0,1091	
ILC-2 KLRG1+	P2Et	25	0,1782	0,1933	0.316	7	0,2279	0,1706	0.123
	Placebo	22	0,1139	0,1314		15	0,1450	0,2717	
ILC-2 CD69+	P2Et	23	0,0023	0,0056	0.972	9	0,0000	0,0000	0.794
	Placebo	21	0,0020	0,0045		13	0,0039	0,0140	
ILC-2 PD1+	P2Et	25	0,0042	0,0093	***0.032****	6	0,0049	0,0120	0.747
	Placebo	21	0,0000	0,0000		16	0,0023	0,0092	
ILC-2 ICOS+	P2Et	25	0,0898	0,1046	0.868	6	0,0714	0,0711	0.693
	Placebo	21	0,0741	0,0630		16	0,1206	0,3026	
ILC-1	P2Et	23	0,2649	0,4121	0.555	9	0,3162	0,4062	0.072
	Placebo	22	0,2669	0,3642		14	0,1386	0,1348	
ILCp	P2Et	23	0,1994	0,3735	0.892	9	0,2769	0,4987	***0.039****
	Placebo	22	0,1445	0,1733		14	0,0575	0,0321	
NK^{Br}	P2Et	25	21,3700	23,2617	0.570	10	28,9826	15,2561	0.060
	Placebo	23	16,3404	14,5166		17	19,7127	22,2201	
NK^{Br} CD25+	P2Et	26	0,2783	0,4207	0.285	11	0,2881	0,3102	0.148
	Placebo	24	0,4506	0,9974		16	0,7977	1,2006	
NK^{Br} CD27+	P2Et	27	3,2033	5,7286	0.386	11	4,0300	4,5694	0.318
	Placebo	23	6,0559	11,9023		16	31,8970	121,8629	
NK^{Br} KLRG1+	P2Et	26	5,1461	5,9508	0.936	11	13,1188	14,1054	0.212
	Placebo	23	6,2407	9,1730		16	12,3504	27,7466	
NK^{Br} CD69+	P2Et	26	0,6453	0,7472	0.936	11	1,1474	2,0133	0.904
	Placebo	23	0,7096	0,8161		16	1,4195	3,4109	
NK^{Br} PD1+	P2Et	26	0,1279	0,1369	0.130	10	0,1797	0,2150	0.623
	Placebo	23	0,2537	0,3441		16	0,2580	0,3065	

NK^{Br} ICOS+	P2Et	27	4,6586	8,1364	0.419	11	10,9111	16,2569	***0.001****
	Placebo	23	2,1641	2,5322		16	2,4126	1,9967	
NK^{Dim}	P2Et	24	167,1915	132,5507	0.625	11	99,4601	83,1331	0.251
	Placebo	23	183,7646	179,0077		16	155,0918	127,2832	
NK^{Dim} CD25+	P2Et	25	0,2904	0,6581	0.808	10	0,0000	0,0000	***0.026****
	Placebo	23	0,4356	0,8700		15	0,3405	0,7917	
NK^{Dim} CD27+	P2Et	26	6,0914	12,1180	0.936	11	22,7743	60,2912	0.458
	Placebo	23	4,5833	6,8268		17	4,6089	5,3559	
NK^{Dim} KLRG1+	P2Et	26	58,8713	40,6412	0.435	11	49,1238	68,5514	0.148
	Placebo	23	72,0073	90,8218		16	106,1035	164,6925	
NK^{Dim} CD69+	P2Et	26	3,3936	4,9547	0.857	11	2,0549	1,5283	0.645
	Placebo	23	4,1022	6,2320		16	3,6515	5,2217	
NK^{Dim} PD1+	P2Et	26	0,6774	1,4248	0.888	11	0,9149	1,1929	1.000
	Placebo	23	0,5081	0,5882		16	0,5891	0,8319	
NK^{Dim} ICOS+	P2Et	26	12,3721	39,2897	0.235	11	0,1125	0,1597	0.512
	Placebo	23	7,7739	24,7313		16	1,7690	4,0068	
CD8+	P2Et	30	297,0460	210,4126	0.794	10	269,6933	143,0864	0.330
	Placebo	24	346,4417	360,5734		19	209,5595	155,6455	
CD8+CD25+	P2Et	31	1,5313	1,7461	0.486	10	6,1533	15,9974	0.377
	Placebo	24	1,8467	1,9546		19	4,0171	11,1159	
CD8+CD27+	P2Et	30	155,0303	117,5499	0.944	10	166,0906	98,0746	0.330
	Placebo	24	347,6050	893,4184		19	126,8121	88,1942	
CD8+KLRG1+	P2Et	30	182,3384	173,1423	0.931	10	144,1296	88,4939	0.573
	Placebo	24	245,1351	303,6461		19	134,2493	116,9982	
CD8+CD69+	P2Et	30	5,7822	4,4900	0.972	11	3,4043	1,5421	0.287
	Placebo	24	15,0724	29,0328		19	7,2532	12,5168	
CD8+PD1+	P2Et	30	38,0477	36,7971	0.725	10	24,3664	16,6163	0.980
	Placebo	22	36,6062	37,1003		17	36,7075	43,2632	
CD8+ ICOS+	P2Et	30	12,0806	11,0865	0.630	10	10,4626	5,9123	0.386
	Placebo	22	11,9194	14,5139		17	9,5576	10,5679	
